# HMG-Like DSP1 Mediates Immune Responses of the Western Flower Thrips (*Frankliniella occidentalis*) Against *Beauveria bassiana*, a Fungal Pathogen

**DOI:** 10.3389/fimmu.2022.875239

**Published:** 2022-04-05

**Authors:** Shabbir Ahmed, Miltan Chandra Roy, Duyeol Choi, Yonggyun Kim

**Affiliations:** Department of Plant Medicals, College of Life Sciences, Andong National University, Andong, South Korea

**Keywords:** *Frankliniella occidentalis*, immunity, DSP1, pathogenicity, *Beauveria bassiana*

## Abstract

Western flower thrips, *Frankliella occidentalis*, is a serious pest by directly infesting host crops. It can also give indirect damage to host crops by transmitting a plant virus called tomato spotted wilt virus. A fungal pathogen, *Beauveria bassiana*, can infect thrips. It has been used as a biopesticide. However, little is known on the defense of thrips against this fungal pathogen. This study assessed the defense of thrips against the fungal infection with respect to immunity by analyzing immune-associated genes of *F. occidentalis* in both larvae and adults. Immunity-associated genes of western flower thrips were selected from three immunity steps: nonself recognition, mediation, and immune responses. For the pathogen recognition step, dorsal switch protein 1 (DSP1) was chosen. For the immune mediation step, phospholipase A_2_ (PLA_2_) and prostaglandin E_2_ synthase were also selected. For the step of immune responses, two phenoloxidases (PO) genes and four proPO-activating peptidase genes involved in melanization against pathogens were chosen. Dual oxidase gene involved in the production of reactive oxygen species and four antimicrobial peptide genes for executing humoral immune responses were selected. All immunity-associated genes were inducible to the fungal infection. Their expression levels were induced higher in adults than in larvae by the fungal infections. However, inhibitor treatments specific to DSP1 or PLA_2_ significantly suppressed the inducible expression of these immune-associated genes, leading to significant enhancement of fungal pathogenicity. These results suggest that immunity is essential for thrips to defend against *B. bassiana*, in which DSP1 and eicosanoids play a crucial role in eliciting immune responses.

## Introduction

The western flower thrips, *Frankliniella occidentalis*, is a polyphagous insect pest. It causes serious economic damage to crops directly by infesting host plants and indirectly by transmitting viral disease (1). Tomato spotted wilt virus (TSWV), a type species of Tospovirus and the only plant-infecting genus in the virus family of Bunyaviridae, can be effectively transmitted by *F. occidentalis* in a persistent-propagative fashion (2). However, it is difficult to control thrips with conventional synthetic insecticides due to their short life cycle (about 2 weeks), high fecundity, thigmotactic behavior, and insecticide resistance (3).

Alternative control tactics have been introduced to effectively and nonchemically reduce populations of thrips (4, 5). *Beauveria bassiana* is an entomopathogenic fungus and an effective epizootic microorganism against *F. occidentalis* (6). It has been reported that *B. bassiana* application is effective in controlling *F. occidentalis* by reducing 70% of thrips’ population when its spore granules are applied to soil to infect pupae of thrips under greenhouse conditions (7). Furthermore, this fungal treatment can reduce the reproductive potential of thrips survived from exposure to a sublethal dose (8). On the other hand, the fungal pathogenicity is usually attenuated by the attack of various insect immune responses (9, 10). In thrips, immune responses are also likely to be effective in defending them against fungal infections (11).

Insect immunity is innate and only programmed nonself molecular patterns such as peptidoglycans and β-1,3-glycan are induced upon bacterial and fungal infections, respectively (12). These nonself signals are recognized by pattern recognition receptors and then propagated by immune mediators to immune executive tissues such as hemocytes and fat body by exhibiting cellular and humoral immune responses (13). Cellular immune responses including phagocytosis, nodule formation, and encapsulation usually performed by hemocytes are acutely induced upon infections (14). Humoral immune responses then mop up the residual pathogens with toxic chemical reactions by producing various antimicrobial peptides (AMPs) or phenoloxidase to induce melanization (15). A recent discovery of damage-associated molecular pattern (DAMP) adds another type of nonself recognition to the insect immune system (16). Dorsal switch protein 1 (DSP1), an ortholog of vertebrate high mobility group box 1, is localized in the nucleus to modulate gene expression by regulating the binding of transcriptional factors to promoters (17). Upon immune challenge, it is released to plasma to act as a DAMP molecule and activate immune responses in coleopteran and lepidopteran insects (16, 18).

From several RNA-seq analyses, a number of immune-associated genes have been annotated in *F. occidentalis* (19). Indeed, TSWV can activate the immune system of *F. occidentalis* (20). The objective of this study was to determine the immune responses of *F. occidentalis* against *B. bassiana* infection.

## Materials and Methods

### Insect Rearing and Fungal Culture

Adults of western flower thrips (*F. occidentalis*) were obtained from Bio Utility (Andong, Korea) and reared under laboratory conditions (temperature of 27 ± 1°C; photoperiod of 16:8 h (L: D), and relative humidity (RH) of 60 ± 5%). Two larval instars (L1 and L2) and adults were reared on sprouted bean seed kernels. L2 stage and adults < 3 days after adult emergence were used for immune and pathogenicity tests. An entomopathogenic fungus, *B. bassiana*, was cultured in a potato dextrose agar (PDA) plate at 25 ± 1°C and RH of 70 ± 5% with a 16:8 h (L:D) photoperiod for 14 days.

### Chemicals

3-Ethoxy-4-methoxyphenol (EMP) was purchased from Sigma-Aldrich Korea (Seoul, Korea) and dissolved in dimethyl sulfoxide (DMSO). Triton X-100 (*t*-octylphenoxy-polyethoxyethanol) was purchased from Sigma-Aldrich Korea and used as an adjuvant in fungal bioassay analysis. *p*-Bromophenacyl bromide (BPB) and methylarachidonyl fluorophosphate (MAFP) were purchased from Sigma-Aldrich Korea. DAPI (4,6-diamidino-2-phenylindole) was purchased from Thermo Scientific (Steingrung, Dreieich, Germany) and dissolved in DMSO. BZA (benzylideneacetone) was purchased from Sigma-Aldrich Korea and dissolved in DMSO. Phosphate-buffered saline (PBS) was prepared with 100 mM phosphoric acid. Its pH was adjusted to 7.4 using NaOH.

### Bioinformatics and Phylogenetic Analyses of Immune Genes

DSP1 sequence of *F. occidentalis* (*Fo-DSP1*) was obtained from GenBank with an accession number of XP_026278027.1. Sequences of *F. occidentalis* were obtained for secretory phospholipase A_2_ (Fo-sPLA_2_A and Fo-sPLA_2_B), calcium-independent phospholipase A_2_ (*Fo-iPLA_2_A* and *Fo-iPLA_2_B*), microsomal prostaglandin E synthase type 2 (*Fo-PGES2*) and dual oxidase (*Fo-Duox*), phenoloxidase-activating protease (*Fo-PAP2A*, *Fo-PAP2B* and *Fo-PAP3*), phenoloxidase (*Fo-PO1*, *Fo-PO2A* and *Fo-PO2B*), antimicrobial peptide genes (*Fo-Apol*, *Fo-Def*, *Fo-Lyz*, and *Fo-Tra1*) from National Center for Biotechnology Information (www.ncbi.nlm.nih.gov). GenBank accession numbers of these genes and related orthologs are described in **Supplementary Tables S1** **–** **S7**. Phylogenetic analyses were performed using MEGA6.06 and ClustalW programs from EMBL-EBI (www.ebi.ac.uk). Bootstrapping values were obtained with 1,000 repetitions to support branching and clustering. Protein domains were predicted using Prosite (https://prosite.expasy.org/) and SMART search program (http://smart.embl-heidelberg.de/).

### Preparation of Fungal Suspension

Conidial suspension of *B. bassiana* was prepared by scraping the fungal culture from 14 days old PDA medium into an Eppendorf tube containing 1 mL of autoclaved Triton X-100 (0.1%) solution (Duksan Pure Chemicals, Ansan, Korea). Conidia of the suspension were counted using a Neubauer hemocytometer (Marienfeld-Superior, Lauda-Königshofen, Germany) under 40× magnification.

### Pathogenicity of *B. bassiana* to *F. occidentalis*

To assess the pathogenic activity of *B. bassiana*, L2 larvae and adults were fed with different concentrations (1×10^8^, 1×10^7^, 1×10^6^, 1×10^5^, 1×10^4^ conidia/mL) of conidial suspension. Briefly, a piece of sprouted bean seed kernel was dipped in 1 mL of conidial suspension from each concentration for 5 min and kept for 10 min to dry under a clean bench. The aseptic condition was strictly followed throughout the process. After L2 larvae or adults were released into a Petri dish (5 × 2 cm), the Petri dish was sealed with parafilm (Bemis Company, Zurich, Switzerland). These Petri dishes were kept in a desiccator (4202-0000, Bel-Art Products, Pequannock, NJ, USA) with a constant temperature of 25 ± 1°C and 75 ± 5% RH which was maintained using a saturated solution of NaCl according to Winston and Bates (21). Dead insects were counted every 12 h up to 5 days by confirming mycosis development on insect cadaver. Each treatment had three replicates. Each replication used 10 larvae or adults.

### Immune Challenge With *B. bassiana*

Sprouted bean seed kernels were dipped into 1 mL of conidial suspension (1×10^6^ conidia/mL) for 5 min and dried for 10 min under aseptic conditions. They were then placed in Petri dishes (5 × 2 cm), in which test insects were fed. After 3 h, the diet was replaced with the fresh kernel. For RNA extraction, both larvae and adult insects were collected at 6, 12, and 24 h after fresh kernel feeding. Each time point was replicated three times with 100 larvae and 80 adults.

### RNA Extraction, RT-PCR, and RT-qPCR

RNA was extracted from different developmental stages (~100 L2, ~100 pupae, or ~100 adults per sample) of *F. occidentalis* using Trizol reagent (Invitrogen, Carlsbad, CA, USA) according to the manufacturer’s instructions. Extracted RNA was quantified using a spectrophotometer (NanoDrop, Thermo Scientific, Wilmington, DE, USA). RNA extract (100 ng per reaction) was used for cDNA synthesis with an RT-premix (Intron Biotechnology, Seoul, Korea). Synthesized cDNA was used as a template for PCR amplification with gene-specific primers (**Supplementary Table S8**). To determine cDNA integrity, the *elongation factor 1* (*EF1*) gene was used. RT-PCR began with initial heat treatment at 94°C for 5 min followed by 35 cycles of denaturation at 94°C for 1 min, annealing at a specific temperature depending on primers (**Supplementary Table S8**) for 30 s, and extension at 72°C for 1 min. PCR reaction was terminated with a final chain extension step at 72°C for 10 min.

Quantitative PCR (qPCR) was performed using SYBR Green Real-Time PCR master mixture (Toyobo, Osaka, Japan) on a Real-Time PCR System (Step One Plus Real-Time PCR System, Applied Biosystem, Singapore). The reaction mixture (20 μL) contained 10 pmol of primers used in RT-PCR and 80 ng of cDNA template. After activating Hotstart Taq DNA polymerase at 94°C for 5 min, the reaction was amplified with 40 cycles of denaturation at 94°C for 30 s, annealing at a specific temperature depending on primers (**Supplementary Table S8**) for 30 s, and extension at 72°C for 30 s. The expression level of *EF1*, a reference gene, was used to normalize target gene expression levels. Each treatment was replicated with three independent biological sample preparations. Quantitative analysis was performed using the comparative CT (2^-ΔΔCT^) method (22).

### PLA_2_ Enzymatic Activity

A commercial assay kit (sPLA_2_ Assay Kit, Cayman Chemical) was used with diheptanoyl thio-phosphatidylcholine as enzyme-substrate to measure PLA_2_ activities in whole bodies of 100 individuals at each stage (L2 larva, pupa, and adults). Whole body extracts were obtained after homogenizing in 100 mM phosphate-buffered saline (PBS, pH 7.4). For inhibitor assay, protein samples were incubated at 25°C with inhibitors (BPB or MAFP) for 30 min. For the immune challenge, larva and adult insects were challenged with *B. bassiana* (1×10^6^ conidia/mL) as mentioned earlier. Samples were collected at 6 h, 12 h, and 24 h post-treatment. sPLA_2_ enzyme activities were measured using Ellman’s reagent [5,5′-dithio-bis-(2-nitrobenzoic acid), DTNB] to produce 5-thio-2-nitrobenzoic acid. DTNB was prepared at 10 mM in 0.4 M Tris buffer (pH 8.0). Assay buffer used 25 mM Tris (pH 7.5) containing 10 mM CaCl_2_, 100 mM KCl, and 0.3 mM Triton X-100. The reaction mixture (175 μL) contained 10 μL of plasma sample, 10 μL of DTNB, 5 μL of assay buffer (or inhibitor for inhibition assay), and 150 μL of the substrate. All inhibitors were used at 100 µM. For negative controls, the same volume of reaction mixture consisted of 10 μL of DTNB, 15 μL of assay buffer, and 150 μL of the substrate. Absorbance was measured at 405 nm. Absorbance for non-enzymatic blank controls was calculated and subtracted from sample wells. The actual extinction coefficient for DTNB was 10.66 mM^−1^. Specific enzyme activity (pmol/min/μg) was calculated by dividing absorbance change by protein amount used as enzyme source for the reaction. Each treatment was replicated with three biologically independent enzyme preparations using different larval or adult samples. Protein concentration was determined by Bradford (23) assay using bovine serum albumin (BSA) as standard.

## Additive Effect of EMP or BZA on Fungal Pathogenicity to *F. occidentalis*

Sprouted bean seed kernels were dipped into 1 mL of the conidial suspension (1×10^5^ conidia/mL) containing different concentrations of EMP (0, 10, 100, 500, and 1,000 ppm) or BZA (0, 10, 100, 500, and 1,000 ppm) for 5 min and dried at 25°C for 10 min under sterile condition. After 10 min, 10 L2 larvae or adults were released into Petri dishes (5 × 2 cm) containing treated kernels. These treated insects were then incubated in an incubator at 25°C as described above and subjected to mortality recording for 5 days at 24 h interval. Each treatment was replicated three times with each replication using 10 individuals.

### Analysis of Immunosuppressants on Immune Responses of *F. occidentalis*

*B. bassiana* (1×10^5^ conidia/mL) was treated along with EMP (1,000 ppm) or BZA (1,000 ppm). Briefly, sprouted bean seed kernels were dipped into 1 mL of the conidial suspension (1×10^5^ conidia/mL) containing EMP or BZA for 5 min and dried at 25°C for 10 min under sterile condition. These treated kernels were provided for feeding for 3 h. After 3 h of feeding, treated kernels were replaced with fresh kernels. Samples for RNA extraction were collected at 24 h post-treatment. Each treatment was replicated three times with each replicate having 100 insects.

### Immunofluorescence Assay of *F. occidentalis* Midgut

Midgut from each adult was collected onto slide glass containing 10 µL of TC100 insect tissue culture medium (Welgene, Gyeongsan, Korea) and incubated at 25°C in a wet chamber for 10 min. After removing TC100, the midgut was then fixed with 4% formaldehyde for 20 min at room temperature (RT). Fixative was replaced with PBS followed by incubation at 25°C for 10 min. After washing with PBS twice, the midgut was permeabilized with 0.2% Triton X-100 in PBS for 10 min at RT. After washing with PBS thrice, the midgut was blocked with 5% skimmed milk in PBS at RT for 20 min. After washing with PBS once, the midgut was incubated with 10 µL of primary antibody (16) raised against Se-DSP1 in rabbit which was diluted in 3% BSA in PBS (1:100) at RT for 1 h 20 min. After washing with PBS thrice, the midgut was incubated with 10 µL of FITC-tagged anti-rabbit secondary antibody (Sigma-Aldrich Korea, diluted with 3% BSA in PBS at 1:5,000) at RT for 1 h. After washing with PBS thrice, the midgut was incubated with 10 µL of 4′,6-diamidino-2-phenylindole (DAPI, 1 μg/mL) (Thermo Scientific, Rockford, IL, USA) in PBS at RT for 5 min for nucleus staining. Finally, after washing with PBS thrice, 5 µL of glycerol and PBS (1:1) mixture was added and cover glass was placed on it. It was then observed under a fluorescence microscope (DM2500, Leica, Wetzlar, Germany) at 400 × magnification.

### Statistical Analysis

Data of continuous variables were subjected to one-way analysis of variance (ANOVA) using PROC GLM in the SAS program (24). Means were compared with the least significant difference (LSD) test at Type I error = 0.05. Median lethal concentration (LC_50_) and time (LT_50_) were subjected to Probit analysis using EPA Probit Analysis Program, ver. 1.5 (U.S. Environmental Protection Agency, USA).

## Results

### Larvae and Adults of *F. occidentalis* Are Susceptible to *B. bassiana* Infection

**Figure 1:**
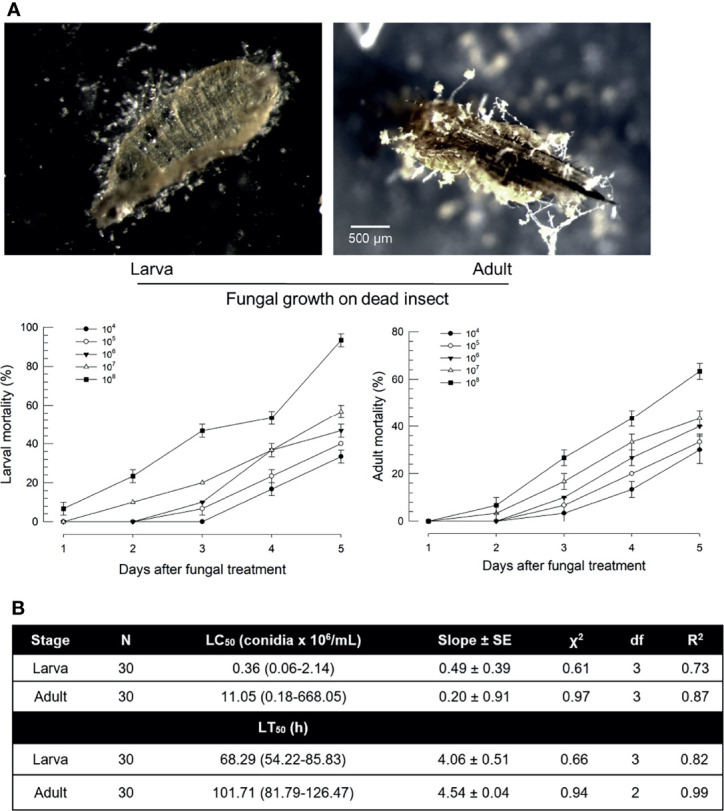
Virulence of *B. bassiana*, an entomopathogenic fungus, to *F. occidentalis*. **(A)** Insecticidal activities of *B. bassiana* at different doses (conidia/mL) to larvae and adults at different time points. Seed kernels were dipped in 1 mL of conidial suspensions from each concentration for 5 min and left to dry at 25°C for 10 min under a clean bench. The photos were taken to confirm the fungal growth from dead insects at 10 days after the fungal treatment. Control without fungi did not show any mortality during this assay. **(B)** Median lethal concentration (LC_50_) and median lethal time (LT_50_) of fungal toxicity. Second instar larvae (L2) and adults were used in this bioassay. To estimate median lethal concentrations (LC_50_s), a fungal concentration of 1×10^6^ conidia/mL was used for each test sample. Median lethal times (LT_50_s) were estimated with the same fungal concentration. An experimental unit (Petri dish) contained 30 larvae or adults. The Petri dish was sealed by parafilm. Each treatment was replicated three times. Both experiments were performed under a constant temperature of 25 ± 1°C with relative humidity (RH) of 75 ± 5% using a saturated solution of NaCl in a desiccator.

Susceptibility of *F. occidentalis* to *B. bassiana* was assessed by oral feeding bioassays using larvae or adults (**Figure 1**). Both developmental stages were susceptible to *B. bassiana*. Dead insects showed sporulation symptoms on body surfaces (**Figure 1A**). The fungal virulence increased in a dose-dependent manner after treatment with fungal spores. However, larvae and adults showed different susceptibilities to *B. bassiana*. Most (> 95%) larvae were dead after treatment with fungal spores at 10^8^ spores/mL. However, only 60% of adults were dead after the same treatment. Median lethal concentrations were different by more than 30 folds between these two developmental stages (**Figure 1B**). In addition, the speed-to-kill was apparently slower for adults by almost two folds than for larvae at the same fungal treatment.

### Fungal Infection Induces Damage-Associated Molecular Pattern (DAMP)

**Figure 2:**
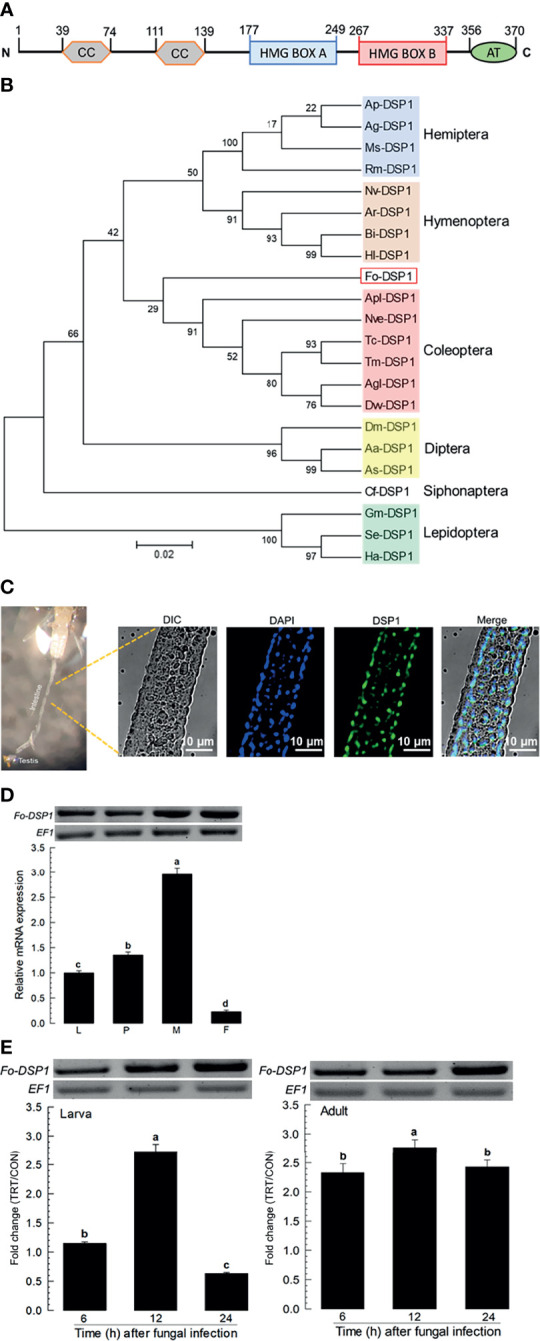
Identification and molecular characterization of DSP1 in *F. occidentalis* (*Fo-DSP1*). **(A)** Domain analysis of *Fo‐DSP1*. Domains were predicted using Prosite (https://prosite.expasy.org/) and SMART protein (http://smart.embl-heidelberg.de/). ‘CC’, ‘HMG’, and ‘AT’ stand for the coiled-coil region, high mobility group, and acidic tail, respectively. **(B)** Phylogenetic analysis of *Fo‐DSP1* with other DSPs from different insect orders. Phylogenetic analysis was performed using MEGA6.06. Bootstrapping values were obtained with 1,000 repetitions to support branching and clustering. Amino acid sequences of DSP1 were retrieved from GenBank with accession numbers shown in **Supplementary Table S1**. **(C)** Localization of Fo-DSP1 in the nuclei of the midgut. Fo-DSP1 and nucleus were stained with antibodies against DSP1 (Green) and DAPI (Blue) against nuclear DNA. Fo-DSP1 was detected with a polyclonal antibody raised against *S. exigua* DSP1. **(D)** Expression of *Fo-DSP1* in different developmental stages of larva (‘L’), pupa (‘P’), male (‘M’), and female (‘F’) adult. **(E)** Inducible expression of *Fo-DSP1* in L2 larvae or adults upon challenge with *B. bassiana* (1×10^6^ conidia/mL) at different time points. RNA samples were collected from the whole body extracts of larvae (~100), pupae (~100), or adults (~100) for each treatment. The expression of an endogenous gene, *EF1*, confirms equal gel loading and integrity of cDNA preparation. Each measurement was replicated three times. Fold changes are calculated by ratios of expression levels of immune-challenges (‘TRT’) over naïve (‘CON’) thrips. Different letters above standard deviation bars indicate significant differences among means at Type I error = 0.05 (LSD test).

The apparent difference between larvae and adults in fungal pathogenicity suggests a differential defense in immune responses of *F. occidentalis*. DSP1, a DAMP molecule in other insects, was suspected to be activated in the gut of *F. occidentalis* in response to an oral infection of *B. bassiana*. Domains of Fo-DSP1 possess HMG Box A and Box B with additional N- and C-terminal extensions (**Figure 2A**). Its predicted amino acid sequence was clustered with other insect DSP1s (**Figure 2B**). Midguts were isolated from adult insects and used for the analysis of Fo-DSP1 localization with a polyclonal antibody raised against Se-DSP1 of *Spodoptera exigua* (**Figure 2C**). The cross-reactivity of the antibody was supported by high sequence homology (69.6%) between *Fo-DSP1* and *Se-DSP1*. Immunofluorescence assay showed that Fo-DSP1 was localized in the nucleus stained with DAPI. *Fo-DSP1* was expressed in larval to adult stages, with adult males showing the highest expression level (**Figure 2D**). Fungal challenge used diet contaminated with *B. bassiana* significantly increased the expression level of *Fo-DSP1* in both larval and adult stages (**Figure 2E**). However, there was a difference in their induction patterns. *Fo-DSP1* was induced only at 12 h after the fungal treatment at the larval stage while it was induced expression constantly from 6 to 24 h at the adult stage.

### Eicosanoid Biosynthesis Is Induced by DSP1 in Response to Fungal Infection

**Figure 3:**
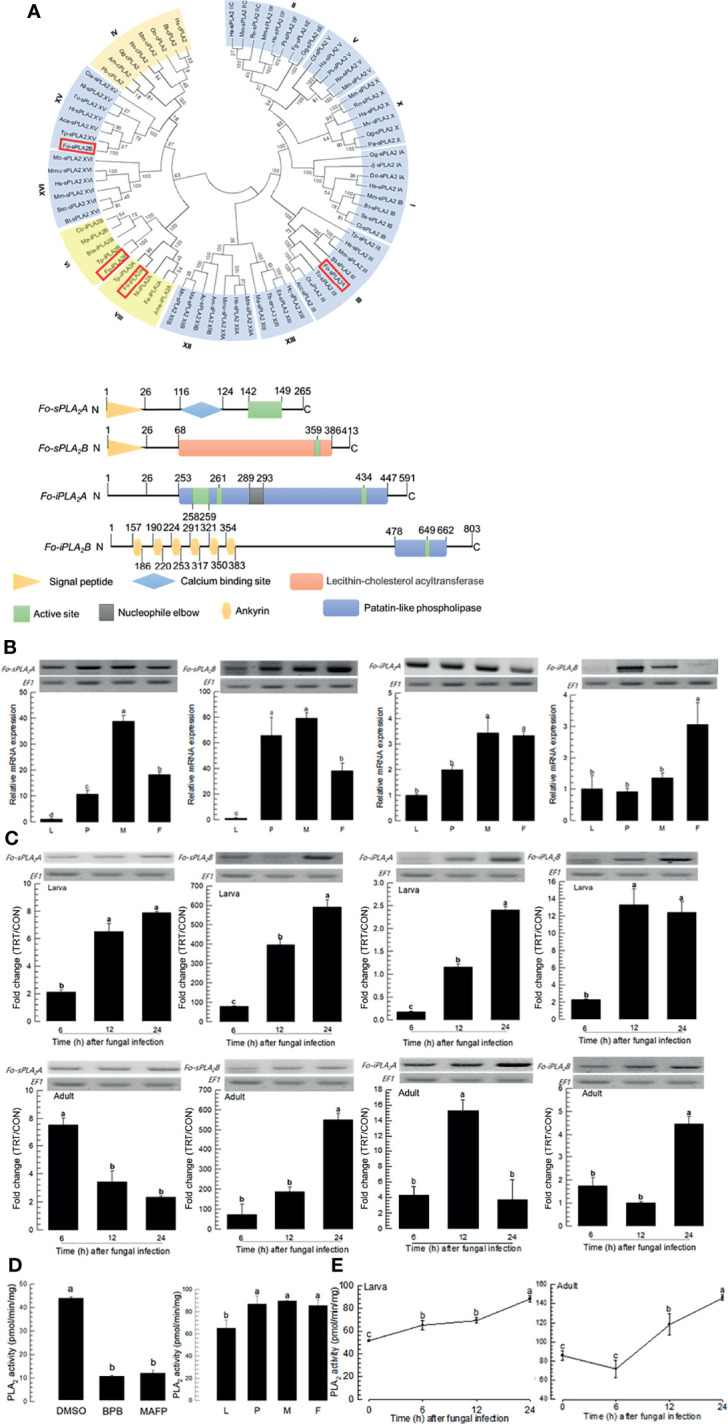
Identification and molecular characterization of two secretory phospholipase A_2_ (sPLA_2_) and two calcium independent PLA_2_ (iPLA_2_) genes in *F. occidentalis* named as *Fo-sPLA_2_A*, *Fo-sPLA_2_B*, *Fo-iPLA_2_A*, and *Fo-iPLA_2_B*. **(A)** Domain and phylogeny analyses of *Fo-PLA_2_* s. Phylogenetic analysis was performed using MEGA6.06. Bootstrapping values were obtained with 1,000 repetitions to support branching and clustering. Amino acid sequences of PLA_2_s were retrieved from GenBank with accession numbers shown in **Supplementary Table S2**. Four *Fo-PLA_2_* s are denoted in the phylogeny tree by rectangular boxes. Domains were predicted using Prosite (https://prosite.expasy.org/) and SMART protein (http://smart.embl-heidelberg.de/). Different colored boxes and shapes represent different regions in the domain. **(B)** Expression of *Fo-PLA_2_* s in different developmental stages of larva (‘L’), pupa (‘P’), male (‘M’), and female (‘F’) adult. **(C)** Inducible expressions of four *Fo-PLA_2_* s (*Fo-sPLA_2_A*, *Fo-sPLA_2_B*, *Fo-iPLA_2_A*, and *Fo-iPLA_2_B*) in L2 larvae or adults upon challenge with *B. bassiana* (1×10^6^ conidia/mL) at different time points. RNA samples were collected from the whole body extracts of larvae (~100) or adults (~100) for each treatment. The expression of an endogenous gene, *EF1*, confirms equal gel loading and integrity of cDNA preparation. Each measurement was replicated three times. Fold changes are calculated by ratios of expression levels of immune-challenges (‘TRT’) over naïve (‘CON’) thrips. **(D)** Enzyme activity of Fo-sPLA_2_. For inhibitor assay, protein samples were collected from the whole body extracts of larvae (~100) for each treatment. Protein samples were incubated with *p*-bromophenacyl bromide (BPB) or methylarachidonyl fluorophosphate (MAFP) *in vitro* for 30 min. To check enzyme activity in naïve condition, the whole body extracts of larvae (~100), pupae (~100), and adults (~100) were used. **(E)** Fo-sPLA_2_ enzyme activity after challenge with *B. bassiana* (1×10^6^ conidia/mL) at different time points. Protein samples were collected from the whole body extracts of larvae (~100) or adults (~100) for each treatment. Each treatment was replicated with three biologically independent enzyme preparations using different larval or adult samples. Different letters above standard deviation bars indicate significant differences among means at Type I error = 0.05 (LSD test).

DSP1 can mediate immune responses by activating PLA_2_ activity in insects (16, 18). Four PLA_2_ genes were obtained from GenBank (**Figure 3A**). Among 16 gene Groups (‘I-XVI’) of PLA_2_, four PLA_2_s of *F. occidentalis* were separately clustered with Group III (*Fo-sPLA_2_A*), Group XV (*Fo-sPLA_2_B*), Group VI (*Fo-iPLA_2_A*), and Group VIII (*Fo-iPLA_2_B*). Two secretory PLA_2_s (*Fo-sPLA_2_A* and *Fo-sPLA_2_B*) were found to have a signal peptide or Ca^2+^-binding domain while two Ca^2+^-independent cellular PLA_2_s (*Fo-iPLA_2_A* and *Fo-iPLA_2_B*) were not. These two iPLA_2_s were different in the ankyrin repeat domain, which was predicted only in *Fo-iPLA_2_B*.

Four *PLA_2_* s of *F. occidentalis* were expressed in all developmental stages. They were highly expressed in adult stages (**Figure 3B**). The fungal challenge with *B. bassiana* significantly increased expression levels of four *PLA_2_* s in both larval and adult stages (**Figure 3C**). Especially, *Fo-sPLA_2_B* was highly induced by several hundred folds in its expression levels in both larval and adult stages in response to the fungal challenge.

PLA_2_ enzyme activity was analyzed using different PLA_2_ inhibitors: BPB as a specific inhibitor for sPLA_2_ and MAFP as a specific inhibitor for cPLA_2_ (**Figure 3D**). Protein extracts of larvae had PLA_2_ enzyme activities, which were significantly inhibited by both inhibitors. PLA_2_ activities were detected in different developmental stages of *F. occidentalis*. They were increased in both larval and adult stages after the fungal infection (**Figure 3E**). The susceptibility of PLA_2_ enzyme activity to MAFP suggests a presence of cPLA_2_ in *F. occidentalis*, but its genome does not have a typical cPLA_2_ ortholog.

**Figure 4:**
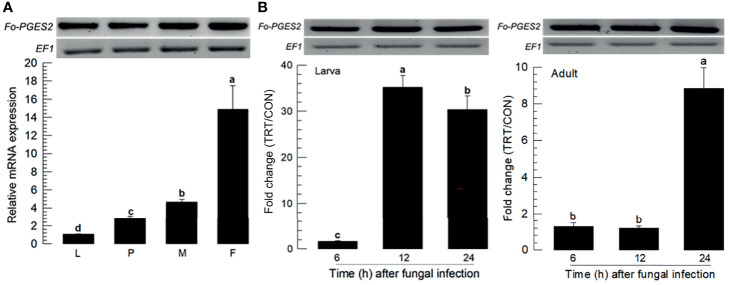
Inducible expression of prostaglandin E2 synthase (PGES2) in *F. occidentalis* (*Fo-PGES2*) upon fungal challenge. **(A)** Expression levels of *Fo-PGES2* in different developmental stages of larva (‘L’), pupa (‘P’), male (‘M’), and female (‘F’) adult. **(B)** Inducible expression of *Fo-PGES2* in L2 larvae or adults upon challenge with *B. bassiana* (1×10^6^ conidia/mL) at different time points. RNA samples were collected from the whole body extracts of larvae (~100), pupae (~100), or adults (~100) for each treatment. The expression of an endogenous gene, *EF1*, confirms equal gel loading and integrity of cDNA preparation. Each measurement was replicated three times. Fold changes are calculated by ratios of expression levels of immune-challenges (‘TRT’) over naïve (‘CON’) thrips. Different letters above standard deviation bars indicate significant differences among means at Type I error = 0.05 (LSD test).

PGE_2_ is one of the PGs commonly detected in different insects (15). *F. occidentalis* ortholog of PGE_2_ synthase 2 (*Fo-PGES2*) was obtained from GenBank (**Table S3**). Its amino acid sequence indicated an enzyme catalytic domain (**Figure S1A**). Phylogenetic analysis indicated that *Fo-PGES2* was clustered with other membrane-bound PGES2 genes (**Figure S1B**). *Fo-PGES2* was expressed in different developmental stages (**Figure 4A**). Its expression was highly inducible in larval and adult stages by the fungal infection (**Figure 4B**).

### Effects of Specific Inhibitors for DSP1 and PLA_2_ on Defense Against Fungal Infection by *B. bassiana*

**Figure 5:**
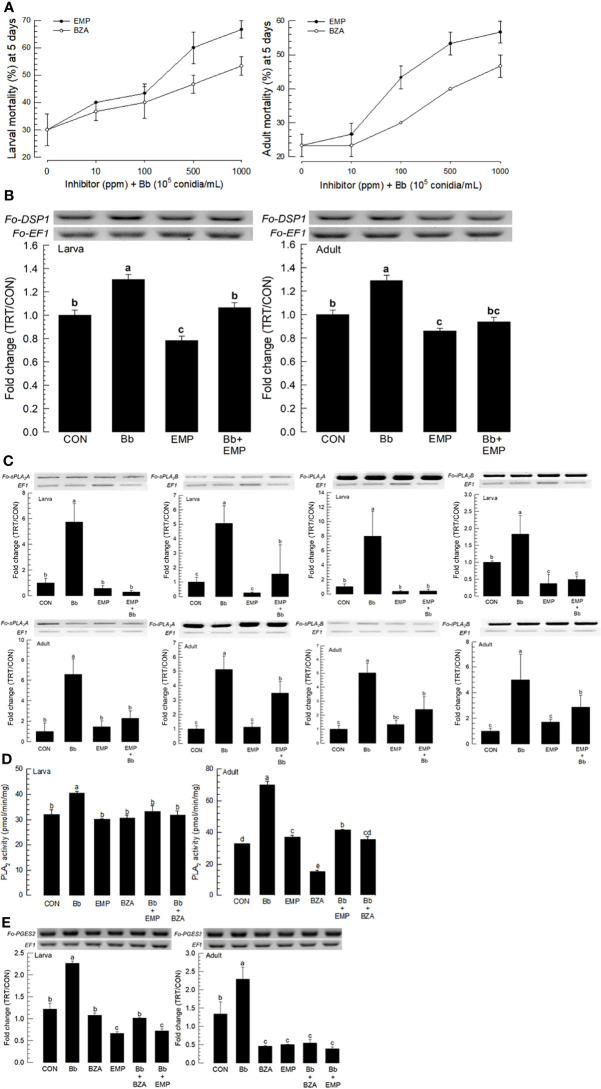
Synergistic effect of EMP or BZA on pathogenicity of *B. bassiana* to *F. occidentalis*. **(A)** Effects of different concentrations of EMP or BZA on the pathogenicity of *B. bassiana* to larvae and adults. Seed kernels were dipped into 1 mL of the conidial suspension (1×10^5^ conidia/mL) containing different concentrations of EMP (0, 10, 100, 500, and 1,000 ppm) or BZA (0, 10, 100, 500, and 1,000 ppm) for 5 min and dried at 25°C for 10 min under sterile condition. Mortality of larvae or adults was recorded for 5 days at 24 h interval. An experimental unit (Petri dish) contained 30 larvae or adults. The Petri dish was sealed with parafilm. Each treatment was replicated three times. Both experiments were performed under a constant temperature of 25 ± 1°C and 75 ± 5% relative humidity (RH) using a saturated solution of NaCl in a desiccator. EMP or BZA alone did not show any mortality at all concentrations in both developmental stages during this assay. **(B)** Suppressive effect of EMP on the induction of *B. bassiana* on the expression of *Fo-DSP1* in larvae and adults. To check immunosuppressive activity, *B. bassiana* (1×10^5^ conidia/mL) along with EMP (1,000 ppm) was used as mentioned previously. Fold changes are calculated by ratios of expression levels of immune-challenges (‘TRT’) over naïve (‘CON’) thrips. **(C)** Suppressive effect of EMP on the induction of *B.bassiana* on the expressions of four *Fo-PLA_2_* s (*Fo-sPLA_2_A*, *Fo-sPLA_2_B*, *Fo-iPLA_2_A*, and *Fo-iPLA_2_B*) in L2 larvae or adults upon challenge with *B. bassiana* in larvae and adults. To check the immunosuppressive activity, *B. bassiana* (1×10^5^ conidia/mL) along with EMP (1,000 ppm) was used as mentioned previously. RNA samples were collected from the whole body extracts of larvae (~100) or adults (~100) for each treatment at 24 h post-infection. **(D)** Inhibitory effect of EMP or BZA on PLA_2_ enzyme activity in larvae or adults. Protein samples were collected from the whole body extracts of larvae (~100) or adults (~100) for each treatment at 24 h post-infection. Each treatment was replicated with three biologically independent enzyme preparations using different larval or adult samples. **(E)** Inhibitory effect of EMP or BZA on the expression of *Fo-PGES2* in larvae or adults. To check immunosuppressive activity, *B.bassiana* (1×10^5^ conidia/mL) along with EMP (1,000 ppm) or BZA (1,000 ppm) was used as mentioned previously. RNA samples were collected from the whole body extracts of larvae (~100) or adults (~100) for each treatment at 24 h post-infection. The expression of an endogenous gene, *EF1*, confirms equal gel loading and integrity of cDNA preparation. Each measurement was replicated three times. Different letters above standard deviation bars indicate significant differences among means at Type I error = 0.05 (LSD test).

EMP is known to bind to and inactivate DSP1 in *S. exigua* and *Tenebrio molitor* (16, 18). BZA is a specific inhibitor for PLA_2_ of insects (25). These two inhibitors were used to treat larvae and adults of *F. occidentalis* along with *B. bassiana* (**Figure 5**). Both inhibitors increased the insecticidal activity of *B. bassiana* to *F. occidentalis* in a dose-dependent manner (**Figure 5A**).

To clarify the effects of inhibitors on the enhancement of fungal pathogenicity, expression levels of *Fo-DSP1*, *Fo-PLA_2_* s, and *Fo-PGES2* were examined after inhibitor treatments. The induction of *Fo-DSP1* after fungal treatment was significantly suppressed by EMP treatment in both developmental stages (**Figure 5B**). *Fo-DSP1* expression after treatment with EMP was also observed in the negative control when EMP alone was used for treatment. Results showed that EMP suppressed the basal level of *Fo-DSP1* in naïve *F. occidentalis*. EMP treatment also suppressed expression levels of all four PLA_2_s (**Figure 5C**). The negative control of PLA_2_ gene expressions by EMP was supported by the suppression of PLA_2_ activities (**Figure 5D**). The increase of PLA_2_ activity after fungal infection was significantly suppressed by the addition of EMP or BZA, a specific PLA_2_ inhibitor. Similarly, BZA or EMP treatment significantly suppressed *Fo-PGES2* expression in both larvae and adults, although the suppression was more prominent in adults than in larvae (**Figure 5E**).

### Eicosanoids Mediate Immune-Associated Oxidases of *F. occidentalis* in Response to the Fungal Infection

**Figure 6:**
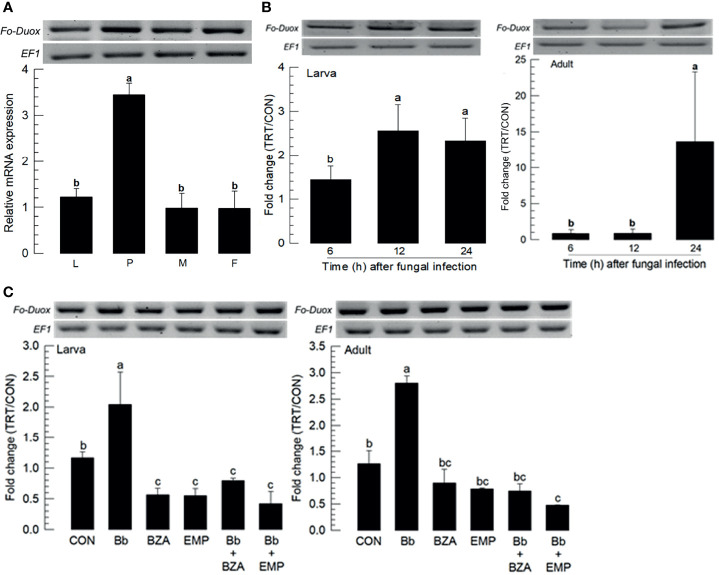
Inducible expression of dual oxidase (Duox) in *F. occidentalis* (*Fo-Duox*) upon fungal challenge. **(A)** Expression levels of *Fo-Duox* in different developmental stages of larva (‘L’), pupa (‘P’), male (‘M’), and female (‘F’) adult. RNA samples were collected from the whole body extracts of larvae (~100), pupae (~100), or adults (~100) for each treatment. **(B)** Inducible expression of *Fo-Duox* in L2 larvae or adults upon fungal challenge with *B. bassiana* (1×10^6^ conidia/mL) at different time points. RNA samples were collected from the whole body extracts of larvae (~100) or adults (~100) for each treatment. **(C)** Inhibitory effects of EMP or BZA on the expression of *Fo-Duox* in larvae or adults. To check this immunosuppressive activity, *B. bassiana* (1×10^5^ conidia/mL) along with EMP (1,000 ppm) or BZA (1,000 ppm) were used as mentioned previously. RNA samples were collected from the whole body extracts of larvae (~100) or adults (~100) for each treatment at 24 h post-infection. The expression of an endogenous gene, *EF1*, confirms equal gel loading and integrity of cDNA preparation. Each measurement was replicated three times. Fold changes are calculated by ratios of expression levels of immune-challenges (‘TRT’) over naïve (‘CON’) thrips. Different letters above standard deviation bars indicate significant differences among means at Type I error = 0.05 (LSD test).

Fungal infection used a feeding method to determine whether *F. occidentalis* might defend the fungal conidia through its gut immunity. Dual oxidase (Duox) plays a crucial role in gut immunity in insects (26). A Duox gene (*Fo-Duox*) was obtained from GenBank (**Table S4**). The additional peroxidase domain in N-terminus supports its identity from NADPH-dependent oxidase (**Figure S2A**). Its predicted amino acid sequences shared homologies with other insect Duox genes (**Figure S2B**). *Fo-Duox* was expressed in different developmental stages (**Figure 6A**). Its expression was highly up-regulated in larvae and adults upon the fungal challenge (**Figure 6B**). However, the induction of this gene was prevented by the addition of an inhibitor specific to DSP1 or PLA_2_ (**Figure 6C**).

**Figure 7:**
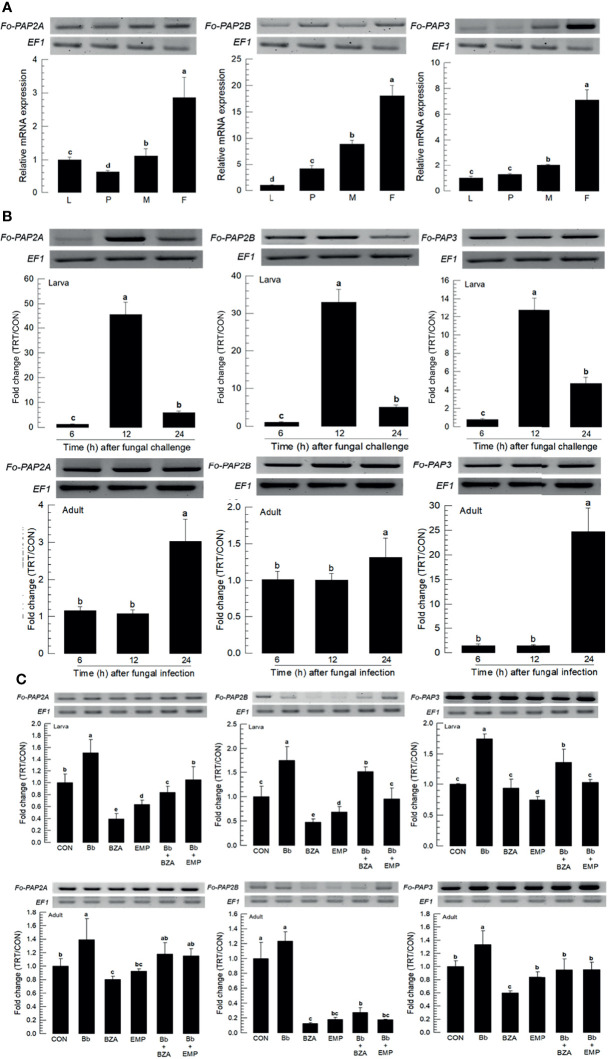
Inducible expression of three prophenoloxidase-activating proteinases (PAPs) in *F*. *occidentalis* (*Fo-PAP*) named *Fo-PAP2A*, *Fo-PAP2B*, and *Fo-PAP3* upon fungal challenge. **(A)** Expression levels of *Fo-PAP* in different developmental stages of larva (‘L’), pupa (‘P’), male (‘M’) and female (‘F’) adult. RNA samples were collected from the whole body extracts of larvae (~100), pupae (~100), or adults (~100) for each treatment. **(B)** Inducible expression of *Fo-PAP* in L2 larvae or adults upon a fungal challenge with *B. bassiana* (1×10^6^ conidia/mL) at different time points. RNA samples were collected from the whole body extracts of larvae (~100) or adults (~100) for each treatment. **(C)** Inhibitory effect of EMP or BZA on the expression of *Fo-PAP* in larvae or adults. To check immunosuppressive activity, *B. bassiana* (1×10^5^ conidia/mL) along with EMP (1,000 ppm) or BZA (1,000 ppm) were used as mentioned previously. RNA samples were collected from the whole body extracts of larvae (~100) or adults (~100) for each treatment after 24 h of post-infection. The expression of an endogenous gene, *EF1*, confirms equal gel loading and integrity of the cDNA preparation. Each measurement was replicated three times. Fold changes are calculated by ratios of expression levels of immune-challenges (‘TRT’) over naïve (‘CON’) thrips. Different letters above standard deviation bars indicate significant differences among means at Type I error = 0.05 (LSD test).

In hemocoel, acute cellular immune responses are likely to be activated by hemocytes with help of the catalytic activity of phenoloxidase (PO), leading to melanization against pathogens (27). PO is produced in an inactive prophenoloxidase (PPO) form, which is cleaved by a specific serine protease called PO-activating protease (PAP) (28). Three PAP genes (*Fo-PAP2A*, *Fo-PAP2B*, and *Fo-PAP3*) were obtained from GenBank (**Table S5**). Domain analyss of these three PAPs possess typical clip domain along with catalytic domain (**Figure S3A**). Different subtypes of PAPs were clustered with their clade members (**Figure S3B**). All three PAPs were expressed in different developmental stages, with adult females having the highest expression levels (**Figure 7A**). Fungal infection induced their expressions, with larvae responding to the fungal infection earlier than adults (**Figure 7B**). The addition of inhibitors specific to DSP1 or PLA_2_ significantly suppressed the up-regulation of the three *PAP*s (**Figure 7C**).

**Figure 8:**
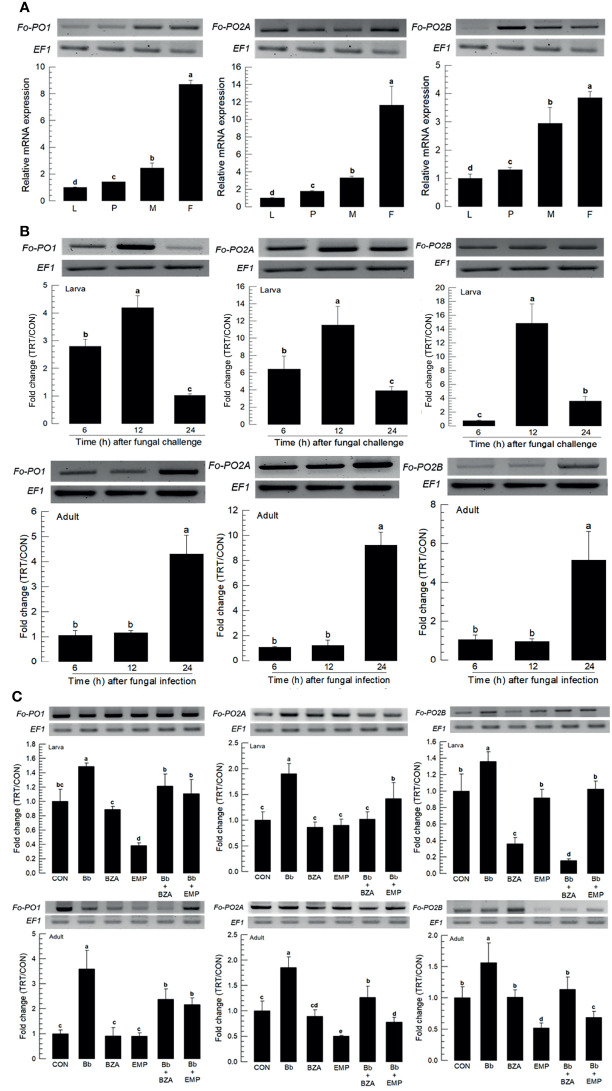
Inducible expression of three prophenoloxidases (PO) in *F. occidentalis* (*Fo-PO*) named *Fo-PO1*, *Fo-PO2A*, and *Fo-PO2B* upon fungal challenge. **(A)** Expression levels of *Fo-PO* in different developmental stages of larva (‘L’), pupa (‘P’), male (‘M’), and female (‘F’) adult. RNA samples were collected from the whole body extracts of larvae (~100), pupae (~100), or adults (~100) for each treatment. **(B)** Inducible expression of *Fo-PO* in L2 larvae or adults upon a fungal challenge with *Beauveria bassiana* (1×10^6^ conidia/mL) at different time points. RNA samples were collected from the whole body extracts of larvae (~100) or adults (~100) for each treatment. **(C)** Inhibitory effect of EMP or BZA on the expression of *Fo-PO* in larvae or adults. To check this immunosuppressive activity, *B. bassiana* (1×10^5^ conidia/mL) along with EMP (1,000 ppm) or BZA (1,000 ppm) were used in a way mentioned previously. RNA samples were collected from the whole body extracts of larvae (~100) or adults (~100) for each treatment after 24 h of post-infection. The expression of an endogenous gene, *EF1*, confirms the equal gel loading and the integrity of the cDNA preparation. Each measurement was replicated three times. Fold changes are calculated by ratios of expression levels of immune-challenges (‘TRT’) over naïve (‘CON’) thrips. Different letters above standard deviation bars indicate significant differences among means at Type I error = 0.05 (LSD test).

Three PO genes (*Fo-PO1*, *Fo-PO2A*, and *Fo-PO2B*) were obtained from GenBank (**Table S6**). Two POs (*Fo-PO1* and *Fo-PO2B*) are likely to be secretory proteins due to their signal peptides in their N termini (**Figure S4A**). However, *Fo-PO2A* appeared to be a transmembrane protein based on domain analysis. A phylogeny tree analysis showed that *Fo-PO2A* and *Fo-PO2B* were closely clustered away from *Fo-PO1* (**Figure S4B**). All three POs were expressed in different developmental stages, with the adult stage having the highest expression levels (**Figure 8A**). Fungal infection induced their expression, with larvae responding to the fungal infection earlier than adults (**Figure 8B**). The addition of an inhibitor specific to DSP1 or PLA_2_ significantly suppressed the up-regulation of these three *PO*s in response to the fungal infection (**Figure 8C**).

### Eicosanoids Induce AMPs of *F. occidentalis* in Response to the Fungal Infection

**Figure 9:**
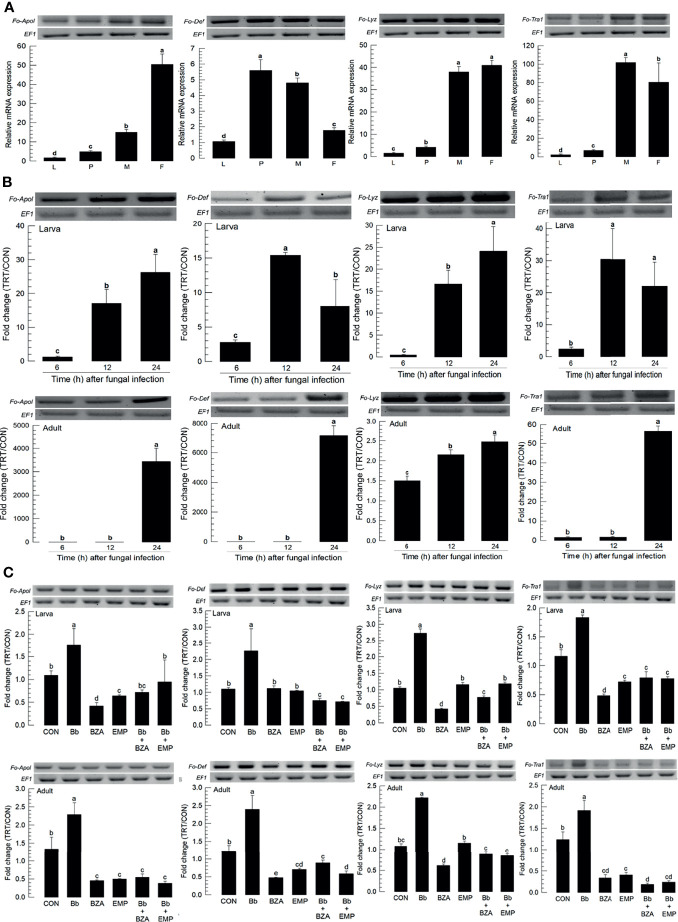
Inhibitory effect of EMP or BZA on the expression of four antimicrobial peptides (AMPs) in *F. occidentalis* named as *Fo-Apol* (apolipophorin III), *Fo-Def* (Defensin), *Fo-Lyz* (lysozyme), and *Fo-Tra1* (transferrin 1). **(A)** Expression of four AMPs in different developmental stages of larva (‘L’), pupa (‘P’), male (‘M’), and female (‘F’) adult. RNA samples were collected from the whole body extracts of larvae (~100), pupae (~100), or adults (~100) for each treatment. **(B)** Inducible expression of four AMPs in L2 larvae or adults upon a fungal challenge with *Beauveria bassiana* (1×10^6^ conidia/mL) at different time points. **(C)** To check this immunosuppressive activity, *B. bassiana* (1×10^5^ conidia/mL) along with EMP (1,000 ppm) or BZA (1,000 ppm) were used in the way mentioned previously. RNA samples were collected from the whole body extracts of larvae (~100) or adults (~100) for each treatment after 24 h of post-infection. The expression of an endogenous gene, *EF1*, confirms the equal gel loading and the integrity of the cDNA preparation. Each measurement was replicated three times. Fold changes are calculated by ratios of expression levels of immune-challenges (‘TRT’) over naïve (‘CON’) thrips. Different letters above standard deviation bars indicate significant differences among means at Type I error = 0.05 (LSD test).

Four AMPs were obtained from GenBank. All AMPs were expressed in different developmental stages, in which most of them except defensin (*Fo-Def*) showed the higher expressions in adults compared to immature stages (**Figure 9A**). All four AMPs were inducible to the fungal infection, in which larvae up-regulated the AMP expressions earlier than adults except lysozyme (**Figure 9B**). The addition of inhibitors specific to DSP1 or PLA_2_ significantly suppressed the up-regulation of these AMPs in response to the fungal infection (**Figure 9C**).

## Discussion

A fungal pathogen, *B. bassiana*, has been widely used as a mycoinsecticide for the biological control of *F. occidentalis* (8). However, little was known on the defense responses of thrips against this fungal infection. Results of this study revealed that fungal infection by *B. bassiana* induced different immune responses of *F. occidentalis*, a worldwide invasive agricultural pest that is now distributed from northern temperate zones to southern temperate zones (1).

Both larvae and adults of *F. occidentalis* were infected by *B. bassiana* after an oral administration using a diet. Especially, larvae were more susceptible to *B. bassiana* than adults. This supports a previous pathogenicity report showing that *B. bassiana* gives serious pathogenicity to different developmental stages of *F. occidentalis*, with the second larval instar being the most susceptible (29). In addition to an insecticidal activity of *B. bassiana*, treatment with *B. bassiana* at a sublethal dose can alter the progeny sex ratio of *F. occidentalis* by producing a male-biased sex ratio (8). This thrips species exhibits an arrhenotokous reproductive mode, which produces males from unfertilized eggs (30). For a practical application, pupae in the soil have been proposed as control targets of *B. bassiana* in a granular formulation, in which fungal conidia can complete processes of surface attachment, germination, and penetration of the body wall of WFT pupa to enter the host within 60 h after treatment (7). These findings suggest that fungal conidia in the gut of *F. occidentalis* might effectively penetrate the gut epithelium and enter the hemocoel to kill both larvae and adults.

Upon the fungal infection, *F. occidentalis* expressed immune responses by activating DSP1, an ortholog of vertebrate HMGB1 known to act as a damage signal in response to pathogen infection in insects (31). HMGB1 is ubiquitously expressed and localized in the nucleus to bind to DNA for regulating gene expression through chromatin remodeling (32). Upon immune challenge, HMGB1 is released passively from dead cells or actively from activated immune cells and acts as a DAMP to activate innate immune responses (33). In insects, DSP1 in the nucleus can act as a corepressor of Dorsal protein in *Drosophila melanogaster* (34). In a mosquito, *Aedes aegypti*, DSP1 can facilitate chromatin remodelling for Toll-associated transcriptional factor to bind to promoter in response to immune challenge (35). In *S. exigua*, DSP1 is released to plasma upon bacterial challenge. It can activate PLA_2_ to mediate various immune responses (16). Later, Mollah et al. (31) showed that DSP1 activates Toll immune signalling to PLA_2_ activation *via* Pelle kinase in *S. exigua*. In *F. occidentalis* in our current study, DSP1 was obtained from its genome and its expression was confirmed in this study. High conserved domain structure and sequence showed that the antibody raised against *S. exigua* DSP1 reacted with DSP1 of *F. occidentalis* and allowed us to observe DSP1 of *F. occidentalis* in its midgut. EMP, a secondary metabolite of a bacterial metabolite of *Xenorhabdus hominickii* (36), is known to inhibit the release of DSP1 in the nucleus (31). When EMP was applied together with the fungal conidia against thrips, it significantly increased the fungal virulence of *B. bassiana* and suppressed other immune responses including PLA_2_ activation. Interestingly, except for adult females, DSP1 expression levels were increased in different development stages of thrips. Although the fungal infection induced DSP1 expression in larvae and adults, relatively lower levels of DSP1 expression in the larvae suggested their higher susceptibility to the fungal infection compared to adults.

Four PLA_2_ genes of *F. occidentalis* were analyzed in this study and classified into secretory (*Fo-sPLA_2_A* and *Fo-sPLA_2_B*) and intracellular (*iPLA_2_A* and *iPLA_2_B*) ones. All four *PLA_2_* s were expressed in larvae and adults of thrips. PLA_2_ has been found in all biological systems. They are classified into at least 16 Groups (I-XIV) based on their amino acid sequences (15). These diverse PLA_2_s are conveniently divided into sPLA_2_, iPLA_2_ (Ca^2+^ independent cellular PLA_2_), and cPLA_2_ (Ca^2+^ dependent cellular PLA_2_). Groups III (*Fo-sPLA_2_A*) and XV (*Fo-sPLA_2_B*) are sPLA_2_s whereas Groups VI (*iPLA_2_A*) and VIII (*iPLA_2_B*) are iPLA_2_s. No cPLA_2_ has been identified in insects. PLA_2_ catalyzes the committed step for eicosanoid biosynthesis (37). Its activation upon fungal infection by *B. bassiana* suggests up-regulation of eicosanoid levels in *F. occidentalis* in larvae and adults. The increase of PLA_2_ activity is likely to be induced by up-regulation of PLA_2_ gene expression and direct activation of its enzyme activity. The immune challenge with bacteria or fungi including *B. bassiana* up-regulates sPLA_2_ and iPLA_2_ gene expressions in *S. exigua* (38, 39). This induction may be associated with Toll immune signaling (27). Indeed, Shafeeq et al. (40) have shown that the Toll immune signaling pathway can activate PLA_2_ probably through phosphorylation using a kinase called Pelle. DSP1 can activate the Toll signal pathway in *S. exigua* (16). These findings suggest that *B. bassiana* fungal infection may trigger the Toll immune signal in *F. occidentalis*, which in turn activates PLA_2_. The activity of PLA_2_ was required for inducing immune responses of *F. occidentalis* in response to *B. bassiana* fungal infection because a specific PLA_2_ inhibitor, BZA, treatment significantly increased the virulence of *B. bassiana* by suppressing immune responses.

Among various eicosanoids, PGE_2_ expression was upregulated in *F. occidentalis* in response to *B. bassiana* fungal infection in this study because *PGES* gene was up-regulated. PGE_2_ is present in diverse insect species. It mediates cellular and humoral immune responses (41). PGE_2_ is especially required for cellular immune responses by stimulating the cytoskeletal rearrangement of actin filaments (42). It can also activate melanization *via* the release of PPO (43). This suggests that up-regulation of PGE_2_ level in response to *B. bassiana* fungal infection can stimulate cellular immune responses by activating hemocytes to perform nodule formation or encapsulation, which is effective in defending insects against fungal infections (27, 44). Melanin formation is required for cellular immune responses through the catalytic activity of PO. Our current study showed an up-regulation of PPO gene expression in response to *B. bassiana* fungal infection.

AMPs and reactive oxygen species (ROS) are two major players in the immune defenses of the insect gut (26). They are likely to be up-regulated in *F. occidentalis* in response to an infection of *B. bassiana* through oral intake. Four different types of AMPs (apolipophorin III, defensin, lysozyme, and transferrin) were also up-regulated upon the fungal infection. In addition, Duox gene expression was highly up-regulated after the fungal infection. Duox catalyzes the production of ROS after being activated by Ca^2+^ (45). PGE_2_ induces Ca^2+^ signals through its specific receptor on the membrane *via* cAMP (42). Especially, the promoter of Duox gene in *S. exigua* has a cAMP response element (CRE) that can bind to CRE-binding protein, which is activated by cAMP in response to PGE_2_ (46). Immune responses of *F. occidentalis* in response to TSWV infection have been analyzed. It has been found that TSWV infection can up-regulate expression levels of lectins for pathogen recognition, Toll with its downstream signal genes, and antimicrobial peptides such as defensin and cecropin) (20). Ogada et al. (47) have analyzed the roles of stress proteins in immune responses of *F. occidentalis* against TSWV infection through a comparative proteomic analysis. TSWV can infect the midgut epithelium of *F. occidentalis* before migrating to the salivary gland for transmission (48). These findings suggest that *F. occidentalis* can recognize the pathogen in the gut upon infection and induce immune responses using AMP and ROS. Our current study suggests that DSP1 can act as a damage signal after *B. bassiana* infection, which activates PLA_2_ *via* Toll signaling. Resulting eicosanoids from the biosynthetic activity of PLA_2_ can lead to cellular and humoral immune responses of *F. occidentalis*.

This study focused on the immune responses of *F. occidentalis* to *B. bassiana* fungal infection. However, behavioral and physiological processes of *F. occidentalis* also play a role in its defense against *B. bassiana* fungal infection. For example, *B. bassiana* infection is known to induce febrile responses in certain host insects, which can reduce the pathogenicity of the fungus (49). Consequently, defensive thermoregulation may decrease the efficacy of *B. bassiana* as a biological control agent (50). Fungal-infected thrips preferentially move to cooler areas while healthy thrips seek out warmer temperatures because their cold-seeking behavior can suppress the growth of *B. bassiana* in infected thrips, thus significantly improving the survivorship of infected thrips (51). Therefore, the defense of *F. occidentalis* against *B. bassiana* fungal infection in diverse physiological processes should be considered.

## Data Availability Statement

The datasets presented in this study can be found in online repositories. The names of the repository/repositories and accession number(s) can be found in the article/**Supplementary Material**.

## Author Contributions

YK contributed to conception and design of the study. SA and MR organized the database. DC performed the statistical analysis. SA wrote the first draft of the manuscript. SA, MR, and YK wrote sections of the manuscript. All authors contributed to manuscript revision, read, and approved the submitted version.

## Funding

This work was carried out with the support of the “Cooperative Research Program for Agriculture Science & Technology Development (Project No. PJ01578901)” funded by the Rural Development Administration, Republic of Korea.

## Conflict of Interest

The authors declare that the research was conducted in the absence of any commercial or financial relationships that could be construed as a potential conflict of interest.

## Publisher’s Note

All claims expressed in this article are solely those of the authors and do not necessarily represent those of their affiliated organizations, or those of the publisher, the editors and the reviewers. Any product that may be evaluated in this article, or claim that may be made by its manufacturer, is not guaranteed or endorsed by the publisher.
