# Supplementary material for: HMG-Like DSP1 Mediates Immune Responses of the Western Flower Thrips (Frankliniella occidentalis) Against Beauveria bassiana, a Fungal Pathogen
Source: Front Immunol. 2022 Apr 5;13:875239. doi: 10.3389/fimmu.2022.875239 (PMC9016178; doi:10.3389/fimmu.2022.875239)
Supplement: Supplementary file 1 [file DataSheet_1.docx]

**Table S1**. GenBank accession number information of sequences used in *Fo-DSP1* phylogeny analysis.

| Sl. No. | Gene name | Acronyms | GenBank Accession |
| --- | --- | --- | --- |
| 1 | *Anopheles stephensi* DSP1 | As-DSP1 | XP 035919508.1 |
| 2 | *Aedes aegypti* DSP1 | Aa-DSP1 | XP_021694839.1 |
| 3 | *Drosophila melanogaster* DSP1 | Dm-DSP1 | AAN09395.1 |
| 4 | *Habropoda laboriosa* DSP1 | Hl-DSP1 | KOC71292.1 |
| 5 | *Nasonia vitripennis DSP1* | Nv-DSP1 | XP_016839160.1 |
| 6 | *Athalia rosae* DSP1 | Ar-DSP1 | XP_012250583.1 |
| 7 | *Bombus impatiens* DSP1 | Bi-DSP1 | XP_003486555.1 |
| 8 | *Rhopalosiphum maidis* DSP1 | Rm-DSP1 | XP_026806372.1 |
| 9 | *Acyrthosiphon pisum* DSP1 | Ap-DSP1 | XP 001944154.1 |
| 10 | *Melanaphis sacchari* DSP1 | Ms-DSP1 | XP_025205315.1 |
| 11 | *Aphis gossypii* DSP1 | Ag-DSP1 | XP 027840861.1 |
| 12 | *Frankliniella occidentalis* DSP1 | Fo-DSP1 | XP_026278027.1 |
| 13 | *Nicrophorus vespilloides* DSP1 | Nve-DSP1 | XP_017786183.1 |
| 14 | *Tribolium castaneum* DSP1 | Tc-DSP1 | XP_973934.2 |
| 15 | *Tenebrio molitor* DSP1 | Tm-DSP1 | MW589636 |
| 16 | *Anoplophora glabripennis* DSP1 | Agl-DSP1 | XP_023310467.1 |
| 17 | *Diabrotica virgifera virgifera* DSP1 | Dvv-DSP1 | XP_028132209.1 |
| 18 | *Agrilus planipennis* DSP1 | Apl-DSP1 | XP_018334345.1 |
| 19 | *Ctenocephalides felis* DSP1 | Cf-DSP1 | XP 026466903.1 |
| 20 | *Galleria mellonella* DSP1 | Gm-DSP1 | XP_026748165.1 |
| 21 | *Spodoptera exigua* DSP1 | Se-DSP1 | MK 737894 |
| 22 | *Helicoverpa armigera* DSP1 | Ha-DSP1 | XP_021183962.1 |

**Table S2**. GenBank accession number information for sequences used in Fo-PLA_2_ phylogeny analysis

| Sl. No. | Gene name | Acronym | Accession number |
| --- | --- | --- | --- |
| 1 | *Homo sapiens* group 1A sPLA_2_ | Hs-sPLA_2_ IA | AAF09020.1 |
| 2 | *Gallus gallus* group IA sPLA_2_ | Gg-sPLA_2_ IA | XP_024999459.1 |
| 3 | *Jaculus jaculus* group 1A sPLA_2_ | Jj-sPLA_2_ IA | XP_004673110.1 |
| 4 | *Dipodomys ordii* group 1A sPLA_2_ | Do-sPLA_2_ IA | XP_012869823.1 |
| 5 | *Mus musculus* group 1B sPLA_2_ | Mm-sPLA_2_ IB | Q9Z0Y2.1 |
| 6 | *Bos taurus* group 1B sPLA_2_ | Bt-sPLA2 IB | P00593.2 |
| 7 | *Suricata suricatta* group 1B sPLA_2_ | Ss-sPLA_2_ IB | XP_029778382.1 |
| 8 | *Canis lapus* group 1B sPLA_2_ | Cl-sPLA_2_ IB | P06596.1 |
| 9 | *Homo sapiens* group IIC sPLA_2_ | Hs-sPLA_2_ IIC | NP_001303651.1 |
| 10 | *Mus musculus* group IIC sPLA_2_ | Mm-sPLA_2_ IIC | NP_032894.2 |
| 11 | *Rattus norvegicus* group IIC sPLA_2_ | Rn-sPLA_2_ IIC | NP_062075.1 |
| 12 | *Fulmarus glacialis* group IIE sPLA_2_ | Fg-sPLA_2_ IIE | KFW01563.1 |
| 13 | *Gallus gallus* group IIE sPLA_2_ | Gg-sPLA_2_ IIE | NP_001171878.1 |
| 14 | *Mus musculus* group IIF sPLA_2_ | Mm-sPLA_2_ IIF | NP_001347804.1 |
| 15 | *Homo sapiens* group IIF sPLA_2_ | Hs-sPLA_2_ IIF | NP_001347798.1 |
| 16 | *Pan troglodytes* group IIF sPLA_2_ | Pt-sPLA_2_ IIF | XP_001163707.1 |
| 17 | *Bos taurus* group III sPLA_2_ | Bt-sPLA_2_ III | NP_001074379.2 |
| 18 | *Homo sapiens* group III sPLA_2_ | Hs-sPLA_2_ III | NP_056530.2 |
| 19 | *Mus musculus* group III sPLA_2_ | Mm-sPLA_2_ III | NP_766379.2 |
| 20 | *Tribolium castaneum* group III sPLA_2_ | Tc-sPLA_2_ III | XP_008201526.1 |
| 21 | *Frankliniella occidentalis* group III sPLA_2_A | Fo-sPLA_2_ A | XP_026271538.1 |
| 22 | *Thrips palmi* group III sPLA_2_ | Tp-sPLA_2_ III | XP_034251452.1 |
| 23 | *Osmia lignaria* group III sPLA_2_ | Ol-sPLA_2_ III | XP_034181167.1 |
| 24 | *Apis cerana cerana* Group III sPLA_2_ | Acc-sPLA_2_ III | PBC28326.1 |
| 25 | *Homo sapiens* group V sPLA_2_ | Hs-sPLA_2_ V | EAW94913.1 |
| 26 | *Rattus norvegicus* group V sPLA_2_ | Rn-sPLA_2_ V | AAT68714.1 |
| 27 | *Pan troglodytes* group V sPLA_2_ | Pt-sPLA_2_ V | JAA22715.1 |
| 28 | *Mus musculus* group V sPLA_2_ | Mm-sPLA_2_ V | AAD45807.1 |
| 29 | *Camelus ferus* group V sPLA_2_ | Cf-sPLA_2_ V | EQB78640.1 |
| 30 | *Mus musculus* group X sPLA_2_ | Mm-sPLA_2_ X | NP_036117.1 |
| 31 | *Gallus gallus* group X sPLA_2_ | Gg-sPLA_2_ X | NP_001171686.1 |
| 32 | *Homo sapiens* group X sPLA_2_ | Hs-sPLA_2_ X | NP_003552.1 |
| 33 | *Manacus vitellinus* Group X sPLA_2_ | Mv-sPLA_2_ X | KFW79807.1 |
| 34 | *Pygoscelis adeliae* Group X sPLA_2_ | Pa-sPLA_2_ X | KFW67599.1 |
| 35 | *Rattus norvegicus* group X sPLA_2_ | Mm-sPLA_2_ X | NP_058872.1 |
| 36 | *Macaca mulatta* group XIIA sPLA_2_ | Mmu-sPLA_2_ XIIA | NP_001181806.1 |
| 37 | *Mus musculus* group XIIA sPLA_2_ | Mm-sPLA_2_ XIIA | NP_075685.2 |
| 38 | *Homo sapiens* group XIIA sPLA_2_ | Hs-sPLA_2_ XIIA | NP_110448.2 |
| 39 | *Atrichornis clamosus* Group XIIB sPLA_2_ | Ac-sPLA_2_ XIIB | NXY18555.1 |
| 40 | *Motacilla alba* Group XIIB sPLA_2_ | Ma-sPLA_2_ XIIB | NWS07230.1 |
| 41 | *Menura novaehollandiae* Group XIIB sPLA_2_ | Mn-sPLA_2_ XIIB | NXE93014.1 |
| 42 | *Alligator mississippiensis* group XIIB sPLA_2_ | Am-sPLA_2_ XIIB | KYO42405.1 |
| 43 | *Histoplasma capsulatum* group XIII sPLA_2_ | Hc-sPLA_2_ XIII | EER41033.1 |
| 44 | *Metarhizium acridum* group XIII sPLA_2_ | Ma-sPLA_2_ XIII | EFY85256.1 |
| 45 | *Tuber borchii* group XIII sPLA_2_ | Tb-sPLA_2_ XIII | AAF80454.1 |
| 46 | *Emmonsia sp* group XIII sPLA_2_ | Es-sPLA_2_ XIII | OAX83076.1 |
| 47 | *Frankliniella occidentalis* group XV sPLA_2_B | Fo-sPLA_2_ XV | XP_026293214.1 |
| 48 | *Thrips palmi* group XV sPLA_2_ | Tp-sPLA_2_ XV | XP_034231554.1 |
| 49 | *Tribolium castaneum* group XV sPLA_2_ | Tc-sPLA_2_ XV | XP_015835827.1 |
| 50 | *Apis cerana* group XV sPLA_2_ | Ace-sPLA_2_ XV | XP_016921550.1 |
| 51 | *Habropoda laboriosa* group XV sPLA_2_ | Hl-sPLA_2_ XV | XP_017799194.1 |
| 52 | *Cimex lectularius* group XV sPLA_2_ | Cle-sPLA_2_ XV | XP_014259587.1 |
| 53 | *Nilaparvata lugens* group XV sPLA_2_ | Nl-sPLA_2_ XV | XP_022196374.1 |
| 54 | *Bos taurus* group XVI sPLA_2_ | Bt-sPLA_2_ XVI | NP_001068748.1 |
| 55 | *Homo sapiens* group XVI sPLA_2_ | Hs-sPLA_2_ XVI | AAI03808.1 |
| 56 | *Mus musculus* group XVI sPLA_2_ | Mm-sPLA_2_ XVI | AAH24581.1 |
| 57 | *Myotis brandtii* group XVI sPLA_2_ | Mb-sPLA_2_ XVI | EPQ02247.1 |
| 58 | *Macaca mulatta* group XVI sPLA_2_ | Mmu-sPLA_2_ XVI | AFE76471.1 |
| 59 | *Sus scrofa* group XVI sPLA_2_ | Ssc-sPLA_2_ XVI | JAA74116.1 |
| 60 | *Rattus norvegicus* cPLA_2_ | Rn-cPLA_2_ | NP_598235.2 |
| 61 | *Python bivittatus* cPLA_2_ | Pb-cPLA_2_ | XP_007435834.1 |
| 62 | *Alligator mississippiensis* cPLA_2_ | Am-cPLA_2_ | KYO24209.1 |
| 63 | *Gallus gallus* cPLA_2_ | Gg-cPLA_2_ | NP_990754.1 |
| 64 | *Bos taurus* cPLA_2_ | Bt-cPLA_2_ | NP_001069332.1 |
| 65 | *Homo sapiens* cPLA_2_ | Hs-cPLA_2_ | NP_077734.1 |
| 66 | *Mus musculus* cPLA_2_ | Mm-cPLA_2_ | NP_032895.1 |
| 67 | *Orcinus orca* cPLA_2_ | Oo-cPLA_2_ | XP_012390439.1 |
| 68 | *Frankliniella occidentalis* iPLA_2_A | Fo-iPLA_2_A | XP_026288906.1 |
| 69 | *Thrips palmi* iPLA_2_A | Tp-iPLA_2_A | P_034246820.1 |
| 70 | *Apis mellifera* iPLA_2_A | Ame-iPLA_2_A | P_026297499.1 |
| 71 | *Formica exsecta* iPLA_2_A | Fe-iPLA_2_A | P_029667145.1 |
| 72 | *Nilaparvata lugens* iPLA_2_A | Nl-iPLA_2_A | P_022202764.1 |
| 73 | *Frankliniella occidentalis* iPLA_2_B | Fo-iPLA_2_B | P_026276941.1 |
| 74 | *Thrips palmi* iPLA_2_B | Tp-iPLA_2_B | P_034231939.1 |
| 75 | *Monomorium pharaonic* iPLA_2_B | Mp-iPLA_2_B | P_012523366.1 |
| 76 | *Cephus cinctus* iPLA_2_B | Bte-iPLA_2_B | P_015588619.1 |
| 77 | *Bombus terrestris* iPLA_2_B | Bte-iPLA_2_B | P_012171186.1 |

**Table S3**. GenBank accession number information of sequences used in *Fo-PGES2* phylogeny analysis.

| Sl. No. | Gene name | Acronyms | GenBank Accession |
| --- | --- | --- | --- |
| 1 | *Frankliniella occidentalis* mPGES2 | Fo-mPGES2 | XM_026420388.1 |
| 2 | *Homo sapiens* mPGES1 | Hs-mPGES2 | NP_004869.1 |
| 3 | *Gallus gallus* mPGES1 | Gg-mPGES1 | ADK66307.1 |
| 4 | *Macaca fascicularis* mPGES1 | Mf-mPGES1 | AAS89037.1 |
| 5 | *Caligus rogercresseyi mPGES1* | Cr-mPGES1 | ACO11433.1 |
| 6 | *Penaeus monodon mPGES1* | Pm-mPGES1 | AFJ11395.1 |
| 7 | *Culex quinquefasciatus mPGES1* | Cq-mPGES1 | XP_001863047.1 |
| 8 | *Pediculus humanus corporis mPGES1* | Phc-mPGES1 | EEB10983.1 |
| 9 | *Homo sapiens* mPGES2 | Hs-mPGES2 | NP_079348.1 |
| 10 | *Gallus gallus mPGES2* | Gg-mPGES2 | XP_415498.1 |
| 11 | *Macaca fascicularis* mPGES2 | Mf-mPGES2 | BAB01608.1 |
| 12 | *Caligus rogercresseyi* mPGES2 | Cr-mPGES2 | ACO11658.1 |
| 13 | *Drosophila melanogaster* mPGES2 | Dm-mPGES2 | NP_524116.2 |
| 14 | *Pediculus humanus corporis* mPGES2 | Phc-mPGES2 | XP_002432321.1 |
| 15 | *Culex quinquefasciatus* mPGES2 | Cq-mPGES2 | XP_001868980.1 |
| 16 | *Bombus terrestris mPGES2* | Bt-mPGES2 | XP_003403370.3 |
| 17 | *Tribolium castaneum mPGES2* | Tc-mPGES2 | XP_973652.1 |
| 18 | *Penaeus monodon mPGES2* | Pm-mPGES2 | AFJ11396.1 |
| 19 | *Gallus gallus* cPGES3 | Gg-cPGES3 | Q90955.1 |
| 20 | *Macaca fascicularis* cPGES3 | Mf-cPGES3 | AAS89038.1 |
| 21 | *Pediculus humanus corporis* cPGES3 | Phc-cPGES3 | XP_002430923.1 |
| 22 | *Penaeus monodon* cPGES3 | Pm-cPGES3 | AFJ11394.1 |
| 23 | *Spodoptera exigua* mPGES2 | Se-mPGES2 | AYD37767.1 |

**Table S4**. GenBank accession number information of sequences used in *Fo-Duox* phylogeny analysis.

| Sl. No. | Gene name | Acronyms | GenBank Accession |
| --- | --- | --- | --- |
| 1 | *Frankliniella occidentalis* Duox | Fo-Duox | XP_026290106.1 |
| 2 | *Aedes aegypti* Duox | Aae-Duox | XP_021700460.1 |
| 3 | *Anopheles darlingi* Duox | Ada-Duox | ETN58940.1 |
| 4 | *Bactrocera dorsalis* Duox | Bdo-Duox | AKS43593.1 |
| 5 | *Ceratitis capitata* Duox | Cca-Duox | XP_004533990.1 |
| 6 | *Lucilia cuprina* Duox | Lcu-Duox | KNC33589.1 |
| 7 | *Zeugodacus cucurbitae* Duox | Zcu-Duox | XP_011189298.1 |
| 8 | *Agrilus planipennis* Duox | Apl-Duox | XP_018323658.1 |
| 9 | *Anoplophora glabripennis* Duox | Agl-Duox | XP_018563303.1 |
| 10 | *Dendroctonus ponderosae* Duox | Dpo-Duox | XP_019766183.1 |
| 11 | *Diabrotica virgifera virgifera* Duox | Dvv-Duox | XP_028130484.1 |
| 12 | *Leptinotarsa decemlineata* Duox | Lde-Duox | XP_023012394.1 |
| 13 | *Onthophagus taurus* Duox | Ota-Duox | XP_022920959.1 |
| 14 | *Cryptotermes secundus* Duox | Cse-Duox | PNF38268.1 |
| 15 | *Zootermopsis nevadensis* Duox | Zne-Duox | XP_021932850.1 |
| 16 | *Formica exsecta* Duox | Fex-Duox | XP_029676591.1 |
| 17 | *Solenopsis invicta* Duox | Sin-Duox | XP_011156000.1 |
| 18 | *Trichoplusia ni* Duox | Tni-Duox | XP_026735041.1 |
| 19 | *Spodoptera litura* Duox | Sl-Duox | XP_022813998.1 |
| 20 | *Papilio machaon* Duox | Pma-Duox | KPJ08497.1 |
| 21 | *Ostrinia furnacalis* Duox | Ofu-Duox | XP_028176585.1 |
| 22 | *Aedes albopictus* Duox | Aal-Duox | XP_019932912.1 |

**Table S5**. GenBank accession number information of sequences used in *Fo-PAP* phylogeny analysis.

| Sl. No. | Gene name | Acronyms | GenBank Accession |
| --- | --- | --- | --- |
| 1 | *Thrips palmi* PAP3 | Tp-PAP3 | XP_034234434.1 |
| 2 | *Bombyx mandarina* PAP2 | Bm-PAP2 | XP_028042038.1 |
| 3 | *Pollicipes pollicipes* PAP2 | Pp-PAP2 | XP_037074844.1 |
| 4 | *Zeugodacus cucurbitae* PAP2 | Zc-PAP2 | XP_011189893.1 |
| 5 | *Ceratina calcarata* PAP2 | Cc-PAP2 | XP_017885598.1 |
| 6 | *Vespa mandarinia* PAP2 | Vm-PAP2 | XP_035724182.1 |
| 7 | *Apis cerana* PAP2 | Ac-PAP2 | XP_016905539.1 |
| 8 | *Drosophila mauritiana* PAP2 | Dm-PAP2 | XP_033174162.1 |
| 9 | *Maniola hyperantus* PAP2 | My-PAP2 | XP_034838304.1 |
| 10 | *Anopheles arabiensis* PAP2 | Aa-PAP2 | XP_040166165.1 |
| 11 | *Thrips palmi* PAP2 | Tp-PAP2 | XP_034235068.1 |
| 12 | *Frankliniella occidentalis* PAP2A | Fo-PAP2A | XP_026283389.1 |
| 13 | *Frankliniella occidentalis* PAP2B | Fo-PAP2B | XP_026283307.1 |
| 14 | *Frankliniella occidentalis* PAP3 | Fo-PAP3 | XP_026272819.1 |
| 15 | *Spodoptera litura* PAP3 | Sl-PAP3 | AAW24481.1 |
| 16 | *Manduca sexta* PAP3 | Ms-PAP3 | AAO74570.1 |
| 17 | *Trichoplusia ni* PAP3 | Tn-PAP3 | XP_026735969.1 |
| 18 | *Galleria mellonella* PAP3 | Gm-PAP3 | XP_026748529.1 |
| 19 | *Plutella xylostella* PAP1 | Px-PAP1 | ANH58161.1 |
| 20 | *Manduca sexta* PAP1 | Ms-PAP1 | AAX18636.1 |
| 21 | *Biston betularia* PAP1 | Bb-PAP1 | ADF43208.1 |

**Table S6**. GenBank accession number information of sequences used in *Fo-PO* phylogeny analysis.

| Sl. No. | Gene name | Acronyms | GenBank Accession |
| --- | --- | --- | --- |
| 1 | *Trichoplusia ni* PO1 | Tn-PO1 | XP_026730296.1 |
| 2 | *Culex quinquefasciatus* PO2 | Cq-PO2 | XP_038104844.1 |
| 3 | *Spodoptera frugiperda* PO1 | Sf-PO1 | XP_035448389.1 |
| 4 | *Anopheles arabiensis* PO2 | Aa-PO2 | XP_040155277.1 |
| 5 | *Tribolium castaneum* PO2 | Tc-PO2 | XP_008194988.1 |
| 6 | *Plutella xylostella* PO2 | Px-PO2 | XP_037974167.1 |
| 7 | *Thrips palmi* PO1 | Tp-PO1 | XP_034256439.1 |
| 8 | *Halyomorpha halys* PO1 | Hh-PO1 | XP_014276959.1 |
| 9 | *Diachasma alloeum* PO1 | Da-PO1 | XP_015123559.1 |
| 10 | *Tribolium castaneum* PO1 | Tc-PO1 | NP_001034493.1 |
| 11 | *Athalia rosae* PO1 | Ar-PO1 | XP_012266497.1 |
| 12 | *Frankliniella occidentalis* PO1 | Fo-PO1 | XP_026272742.1 |
| 13 | *Frankliniella occidentalis* PO2A | Fo-PO2A | XP_026273940.1 |
| 14 | *Frankliniella occidentalis* PO2B | Fo-PO2B | XP_026281547.1 |
| 15 | *Chelonus insularis* PO1 | Ci-PO1 | XP_034943853.1 |
| 16 | *Ctenocephalides felis* PO2 | Cf-PO2 | XP_026465405.1 |
| 17 | *Drosophila serrate* PO2 | Ds-PO2 | XP_020814556.1 |
| 18 | *Hermetia illucens* PO2 | Hi-PO2 | XP_037922217.1 |
| 19 | *Teleopsis dalmanni* PO2 | Td-PO2 | XP_037950695.1 |

**Table S7**. GenBank accession number information of AMP genes used in this study.

| Sl. No. | Gene name | Acronyms | GenBank Accession |
| --- | --- | --- | --- |
| 1 | Apolipophorin III | Fo-Apol | XP_026275826.1 |
| 2 | Defensin | Fo-Def | XP_026284438.1 |
| 3 | Lysozyme | Fo-Lyz | XP_026291031.1 |
| 4 | Transferrin 1 | Fo-Tra1 | XP_026287716.1 |

**Table S8**. Primers used in this study.

| Genes | Orientation | Sequence (5ʹ - 3ʹ) | Uses | Annealing temperature (ºC) | Expected size (bp) |
| --- | --- | --- | --- | --- | --- |
| *EF1* | Forward | TCAAGGAACTGCGTCGTGGAT | RT-qPCR | 52.0 | 130 |
|  | Reverse | ACAGGGGTGTAGCCGTTAGAG |  |  |  |
| *DSP1* | Forward | GCGCCCAAGCGTTCATTATC | RT-qPCR | 55.0 | 195 |
|  | Reverse | GTCCCTCTCATATCGGGCCT |  |  |  |
| *sPLA_2_A* | Forward | GTCTAACCTCCTCAGCTCCA | RT-qPCR | 55.0 | 173 |
|  | Reverse | TCGTCTCTACACCCAAAGG |  |  |  |
| *sPLA_2_B* | Forward | TGTTTGCTACTTCTCGTCGC | RT-qPCR | 55.0 | 324 |
|  | Reverse | TCGTCTCTACACCCAAAGG |  |  |  |
| *iPLA_2_A* | Forward | AAGCTCATTCACACTAGCCG | RT-qPCR | 55.0 | 317 |
|  | Reverse | ACCACACATTGAAGTGGGAC |  |  |  |
| *iPLA_2_B* | Forward | GTCTAACCTCCTCAGCTCCA | RT-qPCR | 55.0 | 195 |
|  | Reverse | TCGTCTCTACACCCAAAGG |  |  |  |
| *PGES2* | Forward | GTGTATGCCTTGGGTGGGAT | RT-qPCR | 52.0 | 150 |
|  | Reverse | GGAAATCTTGCGTGCTGGTG |  |  |  |
| *Duox* | Forward | CAGCAGGTCACCGAACTCAT | RT-qPCR | 52.0 | 193 |
|  | Reverse | AGCTCGTCATTCTCGCTACG |  |  |  |
| *PAP2A* | Forward | ACGGCGAGGGAATTATCGAC | RT-qPCR | 52.0 | 311 |
|  | Reverse | CAACACGTTGCTCTGGTGAC |  |  |  |
| *PAP2B* | Forward | CAACGACAAGAGCCTCCACA | RT-qPCR | 52.0 | 248 |
|  | Reverse | GGCGAGTCTTGCGGAGTAAT |  |  |  |
| *PAP3* | Forward | GGATCTCTCTGCTCGCCATC | RT-qPCR | 52.0 | 133 |
|  | Reverse | GACTCGCGCTACCTGTTCG |  |  |  |
| *PO1* | Forward | TCAACCCCTACCTGTTCGTG | RT-qPCR | 52.0 | 188 |
|  | Reverse | GATGATGATGGGCAGTCGCT |  |  |  |
| PO2A | Forward | GTACCTGTTCGACAGACCCG | RT-qPCR | 52.0 | 287 |
|  | Reverse | CGAAGAAATGCCAGAAGCGG |  |  |  |
| PO2B | Forward | TTCTTCGAAGCGGCAAACAC | RT-qPCR | 52.0 | 162 |
|  | Reverse | GAAGTACTGGGTCTGCTCGG |  |  |  |
| Apol | Forward | ACCGGGCCCCAGGCCTCCCG | RT-qPCR | 52.0 | 133 |
|  | Reverse | CCCTGGCGTCGGCGTCGTCC |  |  |  |
| Def | Forward | ATGCAGAGCCTCACCTTAGG | RT-qPCR | 54.0 | 153 |
|  | Reverse | CCGGGGCGAGCGGCCAGTGC |  |  |  |
| Lyz | Forward | TATGGGAGCGGTGTACTTTA | RT-qPCR | 52.0 | 139 |
|  | Reverse | CCATCGGTGTTTACCGTGTT |  |  |  |
| Tra1 | Forward | GACTTCGGCGTCCTCACCGCG | RT-qPCR | 52.0 | 171 |
|  | Reverse | GCCGCTGAGGAGCCCGGTGT |  |  |  |

**Figure S1.** Identification of prostaglandin E2 synthase (PGES2) in *F. occidentalis* (*Fo-PGES2*). **(A)** Domain analysis of *Fo‐PGES2*. Domains were predicted using Prosite (<https://prosite.expasy.org/>) and SMART protein (<http://smart.embl-heidelberg.de/>). ‘GST’ stands for glutathione s-transferase. **(B)** Phylogenetic analysis of *Fo‐PGES2* with other PGES2s from different insects. Phylogenetic analysis was performed using MEGA6.06. Bootstrapping values were obtained with 1,000 repetitions to support branching and clustering. Amino acid sequences of PGES2 were retrieved from GenBank with accession numbers shown in **Supplementary Table S3**. The following three clusters are shown in the tree: microsomal prostaglandin E synthase 2 (mPGES2), microsomal prostaglandin E synthase 1 (mPGES1), and microsomal prostaglandin E synthase 2 (mPGES3).

**Figure S2.** Identification of dual oxidase (Duox) in *F. occidentalis* (*Fo-Duox*). **(A)** Domain analysis of *Fo‐Duox*. Domains were predicted using Prosite (<https://prosite.expasy.org/>) and SMART protein (<http://smart.embl-heidelberg.de/>). FAD, flavin adenine dinucleotide; NAD, nicotinamide adenine dinucleotide. **(B)** Phylogenetic analysis of *Fo‐Duox* with other Duoxs from different insect orders. Phylogenetic analysis was performed using MEGA6.06. Bootstrapping values were obtained with 1000 repetitions to support branching and clustering. Amino acid sequences of Duox were retrieved from GenBank with accession numbers shown in **Supplementary Table S4**.

**Figure S3.** Identification of three prophenoloxidase-activating proteinases (PAPs) in *F. occidentalis* (*Fo-PAP*) named *Fo-PAP2A*, *Fo-PAP2B*, and *Fo-PAP3*. **(A)** Domain analysis of *Fo‐PAP*. Domains were predicted using Prosite (<https://prosite.expasy.org/>) and SMART protein (<http://smart.embl-heidelberg.de/>). Different colored boxes and shapes represent different regions in the domain. **(B)** Phylogenetic analysis of *Fo‐PAP* with other PAPs from different insects. Phylogenetic analysis was performed using MEGA6.06. Bootstrapping values were obtained with 1000 repetitions to support branching and clustering. Amino acid sequences of PAP were retrieved from GenBank with accession numbers shown in **Supplementary Table S5**.

**Figure S4.** Identification of three prophenoloxidases (PO) in *F. occidentalis* (*Fo-PO*) named *Fo-PO1*, *Fo-PO2A,* and *Fo-PO2B*. **(A)** Domain analysis of *Fo‐PO*. Domains were predicted using Prosite (<https://prosite.expasy.org/>) and SMART protein (<http://smart.embl-heidelberg.de/>). Different colored boxes and shapes represent different regions in the domain. **(B)** Phylogenetic analysis of *Fo‐PO* with other POs from different insects. Phylogenetic analysis was performed using MEGA6.06. Bootstrapping values were obtained with 1000 repetitions to support branching and clustering. Amino acid sequences of PO were retrieved from GenBank with accession numbers shown in **Supplementary Table S6**.


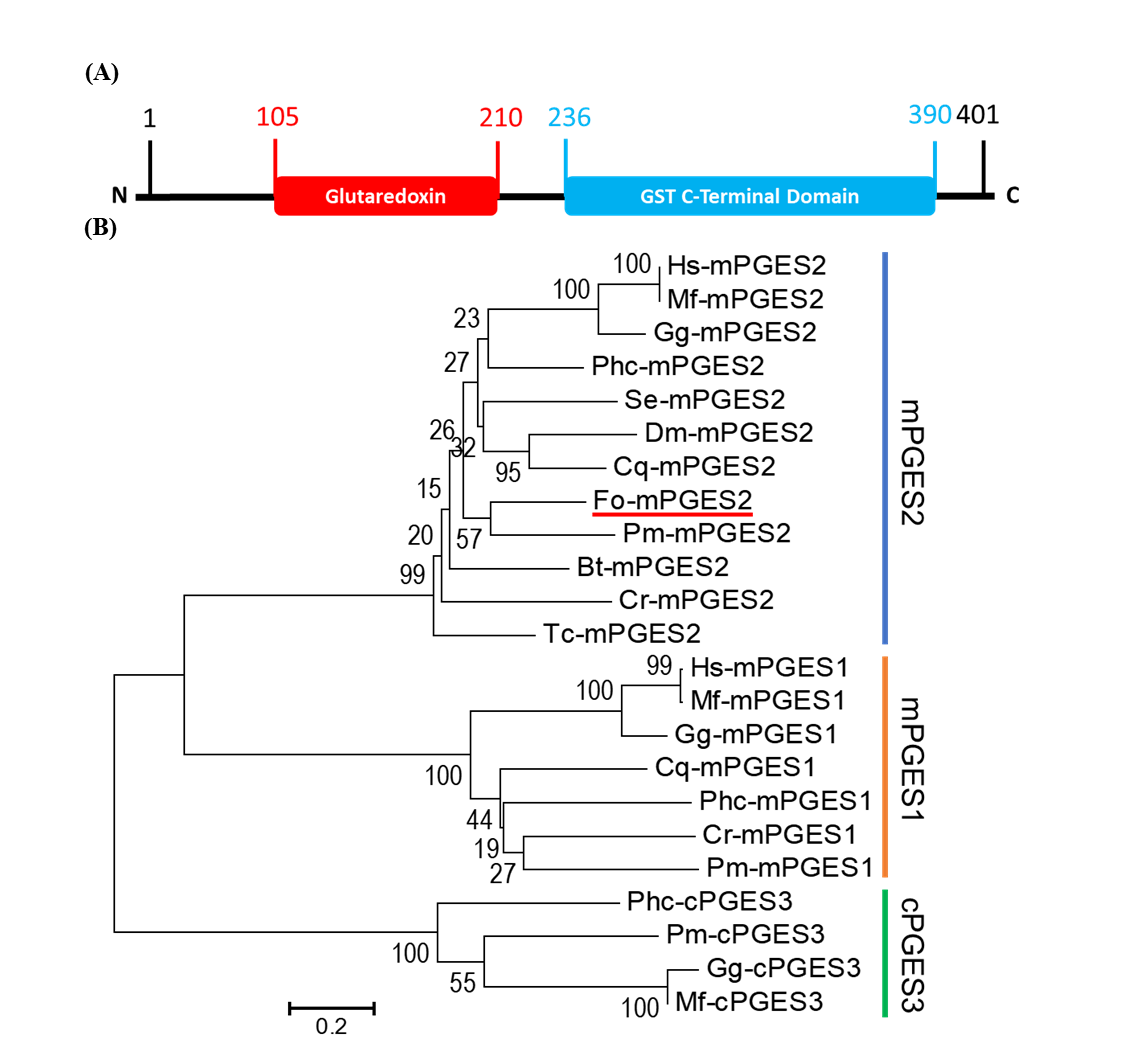


**Figure S1**


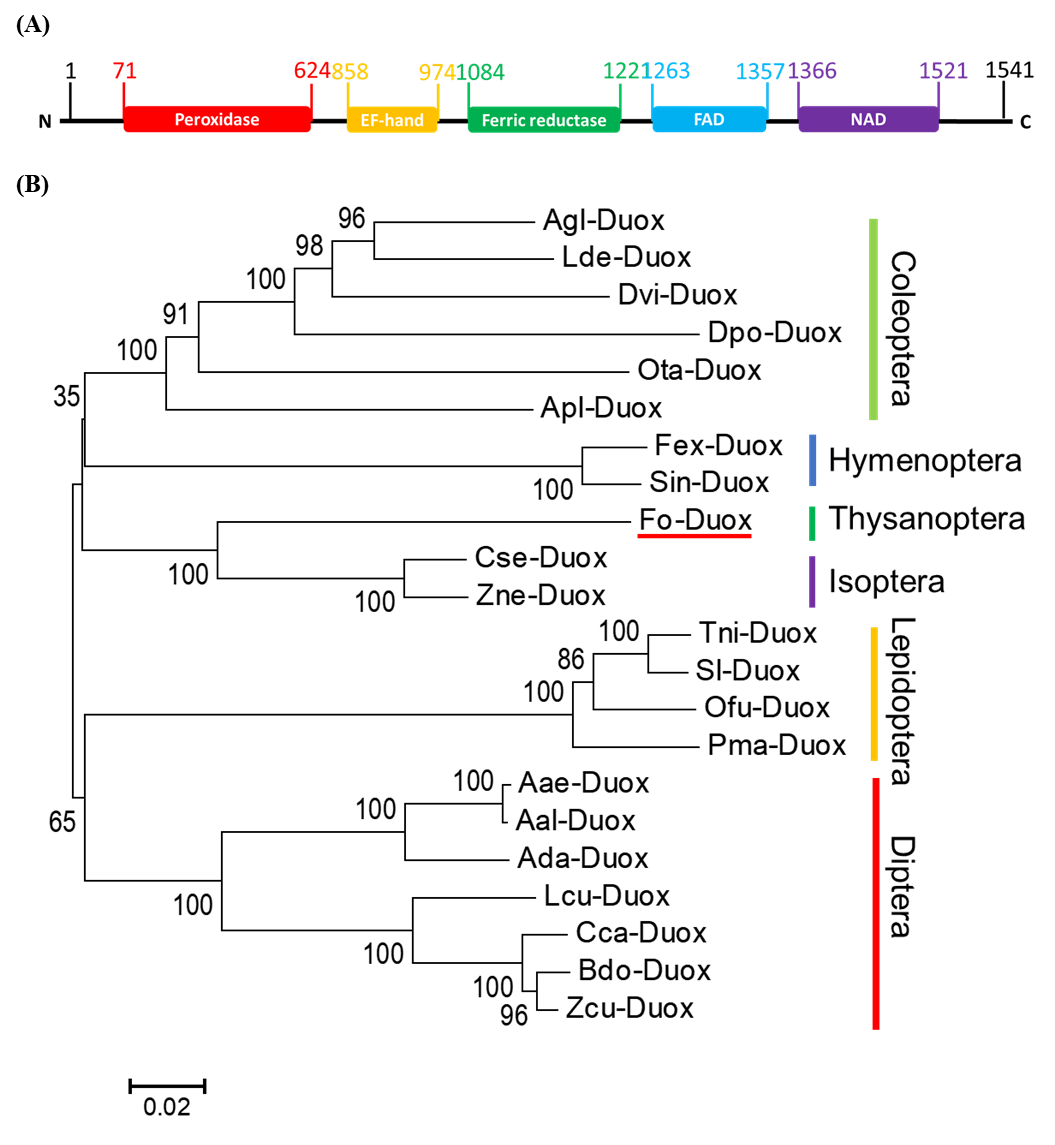


**Figure S2**


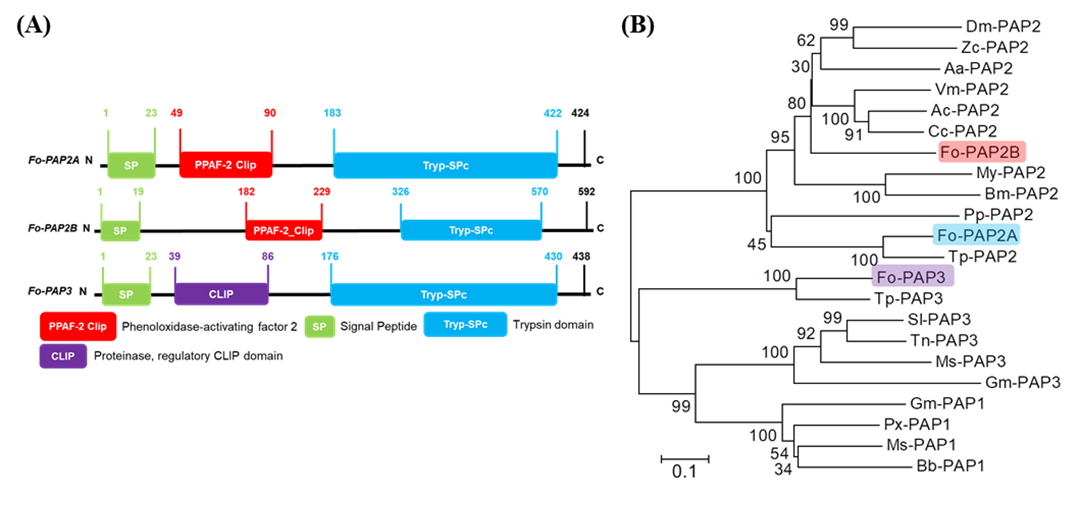


**Figure S3**


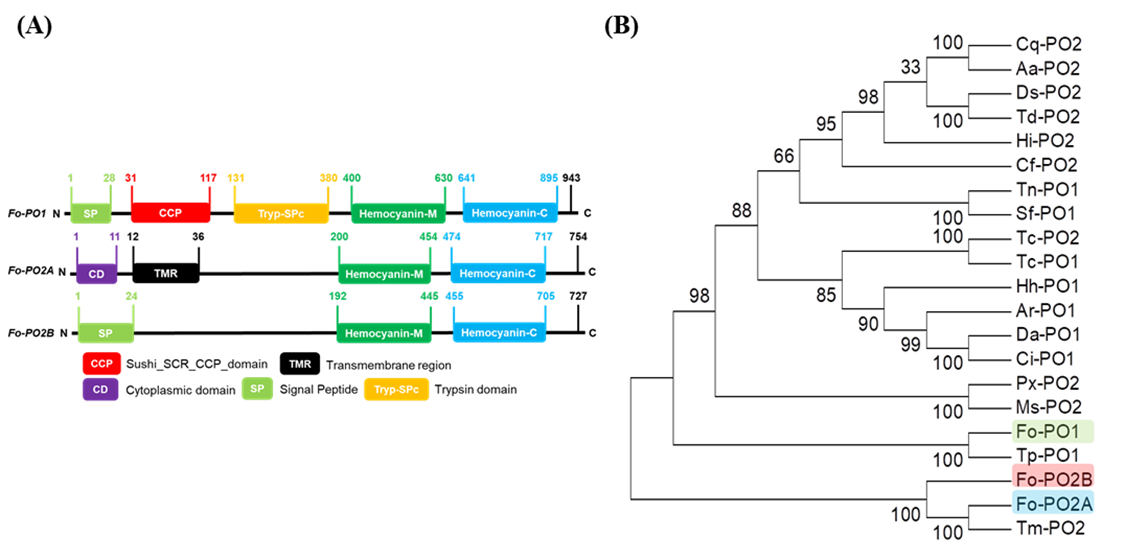


**Figure S4**
